# Seroprevalence of markers and immunization for hepatitis B in community health workers: a scoping review

**DOI:** 10.1590/1980-220X-REEUSP-2025-0358en

**Published:** 2026-03-09

**Authors:** Tauana de Souza Amaral, Sara Oliveira Souza, Márcia Alves Dias de Matos, Gláucia Oliveira Abreu Batista Meireles, Cácia Régia de Paula, Marcos André de Matos

**Affiliations:** 1 Universidade Federal de Goiás, Programa de Pós-Graduação em Enfermagem e Saúde, Goiânia, GO, Brazil.; 2 Universidade Federal de Goiás, Instituto de Patologia Tropical e Saúde Pública, Programa de Pós-Graduação em Biologia das Relações Parasito-Hospedeiro, Goiânia, GO, Brazil.; 3 Associação Educativa Unievangélica, Anápolis, GO, Brazil.; 4 Universidade Federal de Jataí, Jataí, GO, Brazil.

**Keywords:** Community Health Workers, Hepatitis B, Biomarkers, Vaccination, Primary Health Care

## Abstract

**Objective:**

To map the scientific evidence concerning the prevalence of hepatitis B virus (HBV) and immunization among Community Health Workers (CHWs) worldwide.

**Method:**

A scoping review was conducted using the LILACS, Medline (via PubMed), Embase, and Scopus databases, as well as grey literature (Google Scholar, Pro-Quest), employing the recommendations of PRISMA-ScR (extension for scoping review) and the *Joanna Briggs Institute* Manual.

**Results:**

Eleven studies were included, all conducted in Brazil and without parameterization of the outcome measure. The estimated prevalence of HBV infection ranged from 8.2% to 11.1%, while vaccination coverage varied between 41% and 88.8%. Only one study used the anti-HBs serological marker to assess immunization, estimating that 71.6% of community health workers were immunized. Two additional studies were based on self-reported anti-HBs, with prevalences of 30.0% and 30.2%.

**Conclusion:**

The overall estimate of HBV infection prevalence and immunization among community health workers could not be determined due to the scarcity and geographical limitations of existing studies, as well as the measurements deparameterization.

## INTRODUCTION

Hepatitis B virus (HBV) infection represents a global challenge as it is a leading cause of chronic liver disease, leading to approximately 1.1 million deaths annually, primarily due to cirrhosis and liver cancer^(1)^. According to updated estimates from the World Health Organization (WHO), approximately 254 million people worldwide are living with hepatitis B, with a disproportionately high burden on young people and in low- and middle-income countries^(2)^. In this context, WHO emphasizes the urgent need to eliminate hepatitis B as a public health threat by 2030. However, data reveals that, by the end of 2022, only 13% of people living with chronic hepatitis B infection had been diagnosed and approximately 3% had received antiviral therapy^(2)^.

HBV is one of the main etiological agents of occupational infections among healthcare workers (HCWs), due to frequent exposure to blood and other bodily fluids in the workplace^(3,4)^. It is estimated that HCWs are up to four times more likely to get infected with HBV compared to the general population^(5,6)^. Annually, approximately 3 million healthcare workers are exposed to the hepatitis B virus in their work activities, resulting in up to 66,000 HBV infections^(6)^.

Once infected, these workers, besides having the risk of developing the chronic form of the disease, can transmit HBV to patients under their care. This reinforces the importance of this group strictly adhering to precautionary measures, since, when they are properly followed, transmission is rare^(7)^. Due to the increased occupational risk, the Centers for Disease Control and Prevention (CDC) and the WHO recommend HBV vaccination as a pre-exposure prophylaxis measure for all workers in healthcare settings, whether providing direct or indirect care. Furthermore, HBV infection is considered one of the few vaccine-preventable sexually transmitted infections^(3,8)^.

There is already robust evidence regarding the high efficacy and safety of the hepatitis B vaccine, including a significant reduction in cases over the years with the gradual implementation of successful immunization programs^(9,10)^. A global assessment study on vaccination policies have identified that among healthcare professionals, the hepatitis B vaccine is the most widely adopted immunobiological worker in healthcare policies for these workers^(11)^. However, authors emphasize that the increasing number of healthcare professionals who are not immunized and do not respond to vaccination represents a serious public health concern^(4,12)^.

From this perspective, several studies characterize the serological and immunological status of occupational health workers in various work environments, including Primary Health Care (PHC)^(3,4,8,13,14)^. However, knowledge about this topic among Community Health Workers (CHWs) worldwide is still limited^(15,16)^, very possibly resulting from the absence of a universal concept for this category of workers, as well as clarity regarding their roles within PHC healthcare teams^(17)^. Although studies on community health workers are still in their early stages, those identified have shown that this group has work functions that often exceed the limits of their duties, exposing them to biological material^(16,18)^, and has low knowledge about hepatitis B^(19)^. Furthermore, the vast majority of CHWs are young adults, vulnerable to risky sexual behaviors^(20)^.

Given this, we conducted a scoping review to map the scientific evidence on the prevalence of HBV and immunization among community health workers, aiming to assist in the formulation of strategies to ensure the achievement of the Sustainable Development Goals and Universal Health Coverage. Prior to conducting this review, a preliminary search was performed in the PROSPERO, Open Science Framework, Figshare, MEDLINE via PubMed, JBI Evidence Synthesis, and Cochrane Database to ascertain the existence of systematic or scoping reviews on the subject; however, no studies or protocols were found.

## METHOD

### Design of Study

This is a scoping review conducted in accordance with the methodology of *Joanna Briggs Institute (JBI)*(21), registered in *Open Science Framework* (https://doi.org/10.17605/OSF.IO/36YRP), which followed a previously published and peer-reviewed protocol^(22)^. The PRISMA Extension for Scoping Reviews (PRISMA-ScR) was used for transparent reporting of the data^(23)^.

### Research Question

What is the global prevalence of hepatitis B virus among community health workers? What percentage of these workers are immunized?

### Eligibility Criteria

#### Participants

This review considered studies including Community Health Workers as professionals who provide health promotion, prevention, and education services to individuals and families through home visits and support in primary health care services^(24)^.

#### Concept

Serological markers and vaccination were considered in the analysis to identify the serological status of CHWs regarding the hepatitis B virus^(25–27)^:

- Individuals infected with the hepatitis B virus: positive for HBsAg and/or total anti-HBc.
- Immunized individuals: anti-HBs ≥ 10 mIU/ml
- Vaccinated individuals: vaccination report or verification of vaccination records showing three doses of the vaccine against the virus.

#### Context

This review considered studies conducted in facilities providing primary health care, defined as the provision of health actions and services according to the needs of the local population, based on scientific evidence, at low cost, and considered the first step for an individual’s contact with other health care systems^(28)^.

#### Types of Sources of Evidence

Primary articles published in full, in any language, with quantitative, qualitative, or mixed study designs were included as sources of evidence. Preprints, dissertations, theses, and grey literature were also consulted. Reviews were excluded from the study. In addition, a manual search of the reference lists of records eligible for full-text reading was conducted to identify additional articles.

#### Search Strategy

A preliminary search using the keywords “Community Health Workers” and “hepatitis B” was conducted in MEDLINE via PubMed to identify if there were eligible studies on the study topic. After this stage, the databases that would comprise this review were selected. The search strategy was conducted in the Latin American and Caribbean Literature in Health Sciences (LILACS), Medline via PubMed, Embase, and Scopus. The PRISMA-Statement for Reporting Literature Searches in Systematic Reviews (PRISMA-S) extension was used for transparent reporting of the search strategy (Chart 1).

**Chart 1:** Search strategy, conducted on January 28, 2025 – Goiânia, GO, Brazil, 2025.

Database	Search Strategy	Records Found (n)
PubMed/Medline	(“Community Health Workers” OR “Barefoot Doctor” OR “Barefoot Doctors” OR “Community Health Aide” OR “Community Health Aides” OR “Community Health Worker” OR “Family Planning Personnel” OR “Family Planning Personnel Characteristics” OR “Village Health Worker” OR “Village Health Workers” OR “health auxiliary” OR “asha” OR “accredited social health activist” OR “asha workers” OR “auxiliary health worker” OR “community health officers” OR “community health practitioner” OR “Lay health worker” OR “lay health advisor” OR “ lay health advisors” OR “lay health promoter” OR “lay health promoters” OR “lay health workers” OR “lay healthworker”) AND (“Hepatitis B” OR “Hepatitis B Virus Infection” OR “hbv infection” OR “hepatitis b infection” OR “hepatitis b viral infection” OR “hepatitis b virus infection” OR “infection by hbv” OR “infection by hepatitis b virus” OR “serum hepatitis” OR “type b hepatitis” OR “viral hepatitis b” OR “viral hepatitis type b” OR “virus hepatitis type b”)	53
Embase	(‘community health workers’/exp OR ‘community health workers’:ti,kw OR ‘barefoot doctor’/exp OR ‘barefoot doctor’:ti,kw OR ‘barefoot doctors’/exp OR ‘barefoot doctors’:ti,kw OR ‘community health aide’:ti,kw OR ‘community health aides’/exp OR ‘community health aides’:ti,kw OR ‘community health worker’/exp OR ‘community health worker’:ti,kw OR ‘family planning personnel’/exp OR ‘family planning personnel’:ti,kw OR ‘family planning personnel characteristics’/exp OR ‘family planning personnel characteristics’:ti,kw OR ‘village health worker’/exp OR ‘village health worker’:ti,kw OR ‘village health workers’:ti,kw OR ‘health auxiliary’/exp OR ‘health auxiliary’:ti,kw OR ‘asha’:ti,kw OR ‘accredited social health activist’/exp OR ‘accredited social health activist’:ti,kw OR ‘asha workers’/exp OR ‘asha workers’:ti,kw OR ‘auxiliary health worker’/exp OR ‘auxiliary health worker’:ti,kw OR ‘community health officers’/exp OR ‘community health officers’:ti,kw OR ‘community health practitioner’/exp OR ‘community health practitioner’:ti,kw OR ‘lay health worker’/exp OR ‘lay health worker’:ti,kw OR ‘lay health advisor’/exp OR ‘lay health advisor’:ti,kw OR ‘lay health advisors’/exp OR ‘lay health advisors’:ti,kw OR ‘lay health promoter’/exp OR ‘lay health promoter’:ti,kw OR ‘lay health promoters’/exp OR ‘lay health promoters’:ti,kw OR ‘lay health workers’/exp OR ‘lay health workers’:ti,kw OR ‘lay healthworker’/exp OR ‘lay healthworker’:ti,kw) AND (‘hepatitis b’/mj OR ‘hbv infection’/mj OR ‘hepatitis b infection’/mj OR ‘hepatitis b viral infection’/mj OR ‘hepatitis b virus infection’/mj OR ‘infection by hbv’/mj OR ‘infection by hepatitis b virus’/mj OR ‘serum hepatitis’/mj OR ‘type b hepatitis’/mj OR ‘viral hepatitis b’/mj OR ‘viral hepatitis type b’/mj OR ‘virus hepatitis type b’/mj)	21
BVS/Lilacs	(“Community Health Workers” OR “Barefoot Doctor” OR “Barefoot Doctors” OR “Community Health Aide” OR “Community Health Aides” OR “Community Health Worker” OR “Family Planning Personnel” OR “Family Planning Personnel Characteristics” OR “Village Health Worker” OR “Village Health Workers” OR “health auxiliary” OR “asha” OR “accredited social health activist” OR “asha workers” OR “auxiliary health worker” OR “community health officers” OR “community health practitioner” OR “Lay health worker” OR “lay health advisor” OR “ lay health advisors” OR “lay health promoter” OR “lay health promoters” OR “lay health workers” OR “lay healthworker”) AND (“Hepatitis B” OR “Hepatitis B Virus Infection” OR “hbv infection” OR “hepatitis b infection” OR “hepatitis b viral infection” OR “hepatitis b virus infection” OR “infection by hbv” OR “infection by hepatitis b virus” OR “serum hepatitis” OR “type b hepatitis” OR “viral hepatitis b” OR “viral hepatitis type b” OR “virus hepatitis type b”)	2
Web of Science	(TS = (“Community Health Workers” OR “Barefoot Doctor” OR “Barefoot Doctors” OR “Community Health Aide” OR “Community Health Aides” OR “Community Health Worker” OR “Family Planning Personnel” OR “Family Planning Personnel Characteristics” OR “Village Health Worker” OR “Village Health Workers” OR “health auxiliary” OR “asha” OR “accredited social health activist” OR “asha workers” OR “auxiliary health worker” OR “community health officers” OR “community health practitioner” OR “Lay health worker” OR “lay health advisor” OR “ lay health advisors” OR “lay health promoter” OR “lay health promoters” OR “lay health workers” OR “lay healthworker”)) AND TS = (“Hepatitis B” OR “Hepatitis B Virus Infection” OR “hbv infection” OR “hepatitis b infection” OR “hepatitis b viral infection” OR “hepatitis b virus infection” OR “infection by hbv” OR “infection by hepatitis b virus” OR “serum hepatitis” OR “type b hepatitis” OR “viral hepatitis b” OR “viral hepatitis type b” OR “virus hepatitis type b”)	41

An additional search was conducted in the MedRxiv database for preprints, and in Google Scholar, OpenGrey, ProQuest Dissertations and Theses Global, the Catalog of Theses and Dissertations via the Capes Journals portal, and the Brazilian Digital Library of Theses and Dissertations (BDTD) for grey literature. Only the first 200 relevant publications available on Google Scholar were considered^(29)^.

The search was conducted using the following descriptors in English, according to the acronym PCC (Participants: Community Health Workers, Concept: Hepatitis B, Context: Primary Health Care). Since there were different concepts of CHWs in the literature, some keywords (Chart 1) were used to broaden the search^(17,30,31)^. Furthermore, due to the absence of articles identified in the preliminary search using the context acronym (Primary Care), it was not included in the final search. There were no language or year restrictions for the selection of articles.

#### Screening and Selection of Evidence Sources

**Figure 1:**
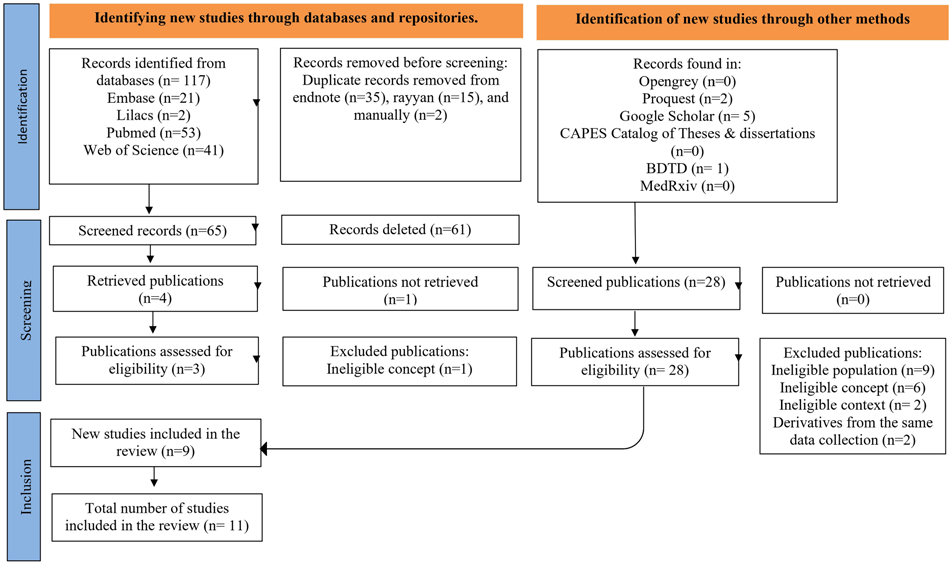
Flowchart for searching, selecting, and including sources of evidence – Goiânia, GO, Brazil, 2025.

The identified citations were combined and uploaded to *EndNote Online* (Clarivate Analytics, PA, USA) for removing duplicates. A pilot test using six references was previously conducted by two reviewers to ensure agreement on the eligibility of the studies. Subsequently, the studies were entered into the Rayyan software for two-step screening. In stage 1, two reviewers (TSA and SOS) independently read the titles and abstracts and selected those that likely met the eligibility criteria. In stage 2, the complete text was examined. The two reviewers held a consensus meeting at each stage, and any disagreements that arose in the selection of records were resolved through discussion or with a third reviewer (MAM). The results of the literature search and the study inclusion process were fully reported in the final scoping review, and a flowchart was included to present the study selection steps (Figure 1).

### Data Extraction

Data extraction was performed by the researcher TSA, and the reviewer SOS verified the described data. An instrument containing information about the publication, study characteristics, study participants, and results was developed for this phase, as suggested by the JBI (Chart 2). Previously, a pilot test was conducted by the reviewers to verify the suitability of the instrument.

**Chart 2:** Synthesis of the studies included in the review – Goiânia, GO, Brazil, 2025.

Citation (author and year)	State/Country	Study objective	Type of study and data collection period	Population (n)	HCW Sample (n)	CHW Sample (n)	Tests/ strategies	Serological markers	HBV prevalence	Vaccination prevalence	Immunization Prevalence	Associated factors
Sanches, 2007^(15)^	Mato Grosso do Sul/Brazil	To assess the immune status of primary healthcare professionals in relation to hepatitis B, as well as to outline the epidemiological factors comprising the representative picture of this disease in these professionals.	Analytical cross-sectional study, conducted between September 2005 and December 2006.	Not specified	347 healthcare workers were surveyed, but for operational reasons, the authors collected 332 blood samples.	59	Commercial kits from the ABBOTT brand using the microparticle enzyme immunoassay methodology.	HBsAg HBeAg anti-HBc anti-HBs anti-HBe	13.5% (95% CI = 4.78%–22.22%)	It does not address	It does not address	It does not address
Garcia and Facchini, 2008^(35)^	Santa Catarina/Brazil	To verify the prevalence of complete hepatitis B vaccination, to stimulate the prevalence of confirmed immunity, and to investigate the factors associated with completing the vaccination schedule among healthcare workers in the municipality of Florianópolis, Santa Catarina, Brazil.	Analytical cross-sectional study, conducted between October and December 2006.	1332	1249	Not specified	Self-report of vaccination	No marker	It does not address	55%	It does not address	It does not address
Assunção et al., 2012^(36)^	Minas Gerais/Brazil	To identify factors associated with hepatitis B vaccination in municipal health workers.	Analytical cross-sectional study, conducted in 2009.	1808	1770	Not specified	Self-report of vaccination	No marker	It does not address	84.1%	It does not address	It does not address
Costa et al., 2013^(37)^	Minas Gerais/Brazil	To investigate the prevalence and factors associated with hepatitis B vaccination among primary healthcare workers, and to analyze the factors considered important in determining hepatitis B vaccination.	Analytical cross-sectional study, conducted in 2009.	797	761	480	Self-report of vaccination	No marker	It does not address	41% (95% CI = 36.59%–45.41%)	It does not address	It does not address
Souza et al., 2015^(38)^	Bahia/Brazil	To assess the prevalence and factors associated with hepatitis B vaccination among Primary Care and Medium Complexity healthcare workers in a municipality in Bahia.	Cross-sectional analytical study conducted in 2011.	707	506	Not specified	Self-report of vaccination	No marker	It does not address	65.3%	It does not address	It does not address
Rezende, 2016^(32)^	Goiás/Brazil	To analyze the biological risk in the work practice of community health workers.	Descriptive cross-sectional study, conducted between September and November 2015.	89	80	80	Vaccination card and self-reported vaccination	No marker	It does not address	82.5% (CI= 74.17–90.83%), however 52.5% had all three doses on their vaccination card, and 30% reported completing the schedule.	30.0% (95% CI = 19.96%–40.04%) through self-reported anti-HBs testing.	It does not address
Souza and Araújo, 2016^(39)^	Bahia/Brazil	To investigate the vaccination status for the four vaccines guaranteed by the National Immunization Program, evaluating the prevalence of vaccination among occupational categories and the completeness of the vaccination schedule among primary care and medium complexity care workers in municipalities of Bahia.	Descriptive cross-sectional study, conducted between 2010 and 2012.	6.191	3.084	Not specified	Self-report of vaccination	No marker	It does not address	88.8%	It does not address	It does not address
Costa et al., 2017^(33)^	Minas Gerais/Brazil	To investigate the prevalence of post-vaccination immunization verification against hepatitis B among primary health care workers and its association with occupational factors, general health conditions, and self-care measures.	Cross-sectional analytical study, data collected between August and December 2011.	797	400	197	Self-reported vaccination and anti-HBs test results.	No marker	It does not address	Not specified	30.2% (95% CI = 23.79%–36.61%), through self-reported anti-HBs testing.	It does not address
Rocha, 2017^(40)^	Minas Gerais/Brazil	To analyze the vaccination status of Community Health Workers in Primary Health Care units in a municipality in the Zona da Mata region of Minas Gerais.	Cross-sectional analytical study, data collected between July and October 2015; and October 2016 to February 2017.	500	400	400	Self-report of vaccination	No marker	It does not address	87.8% (95%CI = 84.59%–91.01%)	It does not address	The time in the occupation of the CHW was associated with the hepatitis B vaccine.
Pinto et al., 2020^(41)^	Espírito Santo/Brazil	To identify the adherence of CHWs to hepatitis B vaccination, to determine the causes of non-adherence to hepatitis B vaccination, and to investigate whether the prevalence of vaccination differs among the surveyed towns.	Analytical cross-sectional study, carried out between April 2016 and April 2017.	664	516	516	Self-report of vaccination	No marker	It does not address	59.7% (95%CI = 55.48%–63.92%)	It does not address	More than five years of experience in the occupation was associated with adherence to hepatitis B vaccination.
Amaral et al., 2023^(16)^	Goiás/Brazil	To identify the vaccination and serological status against hepatitis B among Community Health Workers, to vaccinate against the hepatitis B virus, and to evaluate the immune response of susceptible CHWs.	A descriptive cross-sectional study was conducted between July and December 2019, followed by a cohort study conducted from January to May 2020.	172	172	109	Vaccination record card and serological tests. For the detection of HBsAg, a rapid test from the brand Bioclin, Brazil was used; and for the other anti-HBc and anti-HBs markers, the chemiluminescence method was used (Architec i1000tm, Abbott Diagnostics).	HBsAg anti-HBc anti-HBs	8.2% (95% CI = 4.4%-15.5%)	68.8% (95% CI = 59.6%–76.7%)	71.6% (95% CI = 62.5%–79.2%)	It does not address

### Analysis and Presentation of Results

The included evidence was presented in a summary chart containing a descriptive overview of the study characteristics, as per the data extraction form included in the review protocol^(22)^. Due to the scarcity of studies, two articles were included in this review because of their epidemiological importance, although they did not use marker titration for hepatitis B, but rather self-reporting^(32,33)^. Other forms of visual data presentation were also explored after data extraction and analysis were completed, such as heat maps and waffle charts^(34)^.

## RESULTS

Figure 1 represents the selection, screening, and inclusion processes of the records that comprised the review. The eleven studies included were carried out between 2007 and 2023 and conducted exclusively in Brazil. A total of 9 articles had a descriptive cross-sectional study design^(15,16,32,33,35–39)^, and only 2 analytical cross-sectional articles, both of which found that time in the occupation was statistically associated with hepatitis B vaccination^(40,41)^.

**Figure 2:**
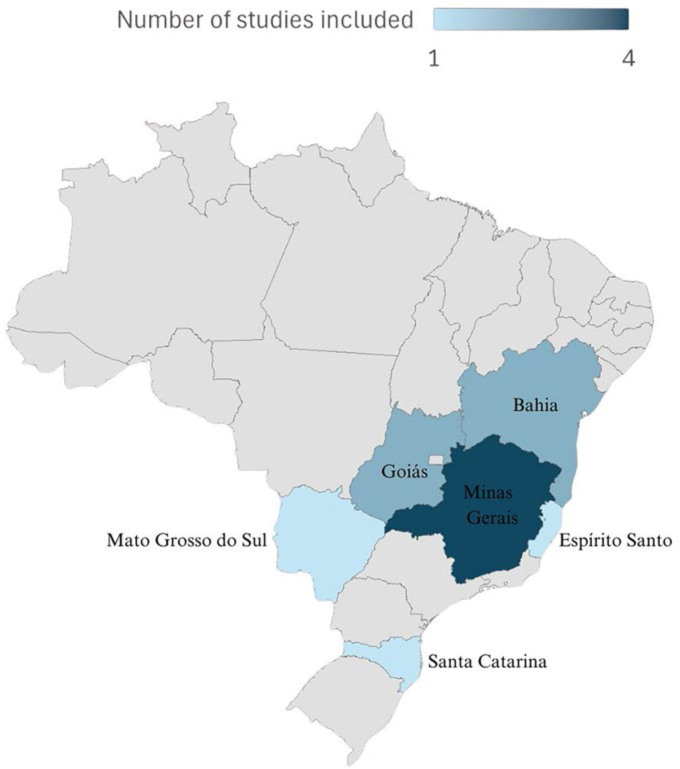
Heat map of the number of studies included by Brazilian state – Goiânia, GO, Brazil, 2025.

Figure 2 shows the heat map of the number of studies included by Brazilian state. It was observed that of the 11 articles, five were conducted in the Southeast region^(33,36,37,40,41)^, three in the Midwest^(15,16,32)^, two in the Northeast^(38,39)^, and one in the South of the Country^(35)^.

It was found that only two studies used serological markers for hepatitis B through blood testing^(15,16)^, one of which was conducted solely with the CHWs^(16)^, in which an overall prevalence of HBV was estimated at 8.2% (95% CI; 4.4%–15.5%)^(16)^. However, the lateral flow immunochromatographic test was used to detect HBsAg. The same investigation also included a cohort study to verify the anamnestic response to quantitative anti-HBs^(16)^. The second study was conducted with all primary healthcare workers and estimated an overall prevalence of hepatitis B of 11.1% (95% CI = 7.72%–14.48%) and 13.5% (95% CI = 4.78%–22.22%) when considering only CHWs^(15)^. This study did not evaluate the immunization status of CHW in isolation, but rather of the entire sample^(15)^.

**Figure 3:**
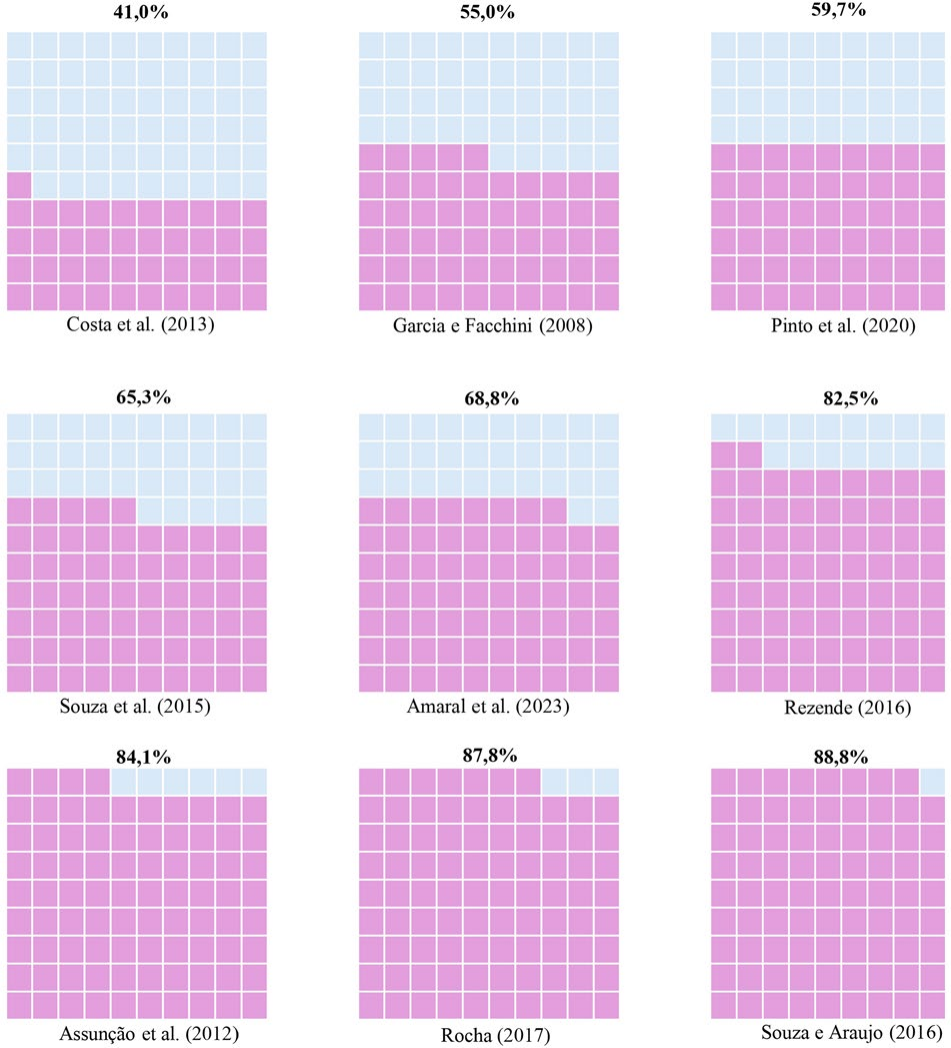
Waffle chart of vaccination prevalence among Community Health Workers found in the studies. The color purple represents the percentage of vaccinated community health workers, while the color blue represents those who are not vaccinated – Goiânia, GO, Brazil, 2025.

Of the total number of articles selected, nine^(16,32,35,36,37,38,39,40,41)^ addressed vaccination among CHWs, with prevalence rates ranging from 41.0% in a study conducted in the city of Montes Claros, Minas Gerais^(37)^ to 88.8% carried out in five cities – Feira de Santana, Itabuna, Jequié, Santo Antônio de Jesus and the Sanitary District of the Historic Center in Salvador, Bahia^(39)^ (Figure 3). Only two articles used the vaccination record card to verify the doses in the vaccination schedule^(16,32)^. The others used self-reporting^(35–41)^.

Regarding the methodology used to identify immunization, only one article used the serological marker for hepatitis B (anti-HBs), estimating a prevalence of 71.6% (95% CI; 62.5%–79.2%) of immunized community health workers^(16)^. Furthermore, two articles used self-reported assessment of this marker, with estimated prevalences of 30.0%^(32)^ and 30.2%^(33)^.

## DISCUSSION

The analysis of the available evidence, as outlined in this scoping review, revealed a scarcity of studies that comprehensively investigate the global prevalence and immunization rates against hepatitis B. Only two studies included in the sample addressed the evaluation of hepatitis B serological markers in community health workers^(15,16)^. However, both lack methodologies based on sensitive laboratory tests for the accurate detection of these markers. Specifically, the study by Amaral et al.^(16)^ used a rapid test to identify the hepatitis B surface antigen (HBsAg); however, the exclusive use of this method may limit diagnostic accuracy, given the possibility of false-positive results. The adoption of confirmatory techniques, such as enzyme immunoassays or molecular tests, would be advisable to ensure greater reliability in the findings. Scientific literature recommends that prevalence and vaccination coverage studies include the evaluation of HBsAg, anti-HBc, and anti-HBs markers using more sensitive tests, to ensure a more accurate assessment of population immunity and virus circulation^(6,27)^.

This finding highlights the deparameterization and lack of knowledge regarding the diagnosis of hepatitis B and serves as an important warning to reinforce the urgency in training healthcare professionals, professional qualification, and the availability of more sensitive laboratory tools^(42)^. Evidence-based clinical practice underscores the need to use a complete panel of serological markers, since the absence of comprehensive testing can lead to false positive or false negative results, unnecessary treatments, and underestimation of population immunity, hindering the identification of susceptible individuals and the implementation of effective preventive measures, which can lead to failures in epidemiological surveillance^(26)^.

Furthermore, nine of the analyzed articles evaluated seroprevalence and immunization against hepatitis B using self-report as a data collection method^(32,33)^. Despite being a practical, low-cost, and easy-to-apply tool, especially in population studies and in contexts where access to vaccination records is restricted, self-reporting has important limitations, such as recall bias, which can compromise the reliability of the results^(6)^. One possible justification for adopting this approach is the limited funding for research, since conducting laboratory tests, although more accurate, involves significantly higher costs compared to using self-reporting.

Available studies indicate that supplementing self-reporting with analysis of vaccination records, such as vaccination record cards, or with laboratory tests, such as the measurement of serological markers, significantly increases the accuracy in assessing vaccination status^(3,43)^. However, access to vaccination records still represents an obstacle for healthcare professionals and researchers, since the general population does not have the habit of considering this document as something essential that should be properly stored^(44)^. In this context, the need to computerize vaccination records becomes evident, although this reality is still far off in many scenarios due to the structural and digital inequalities faced by health units globally.

All the selected studies were conducted in Brazil, most likely because CHWs are considered healthcare professionals^(45)^, which demonstrates the importance of the Brazilian Public Health System. In contrast, in other countries, CHWs often work as volunteers, receive low pay, and lack due professional recognition^(46)^.

Furthermore, this understanding is supported by specific legislation for the category, which establishes fundamental guidelines related to unhealthy working conditions, the performance of periodic occupational health examinations, and the provision and proper use of Personal Protective Equipment (PPE)^(47)^. Additionally, the findings reinforce the ongoing efforts of Brazil’s National Immunization Program (PNI), which plays a crucial role in monitoring and improving immunization rates, as well as contributing to the identification of gaps in local health systems^(48)^.

Brazil’s leadership in implementing vaccination and public health strategies highlights the need to expand international research, allowing for a more comprehensive comparison between different global contexts and solutions.

Most studies on hepatitis B in Brazil have been conducted in the Southeast region^(33,36,37,40,41)^, while states with a higher prevalence of infection, such as those in the Northeast region^(38,39)^, were underrepresented in the scientific literature^(49)^. This disparity underscores inequities in access to healthcare, reflecting structural inequalities that directly impact epidemiological surveillance and the implementation of effective public policies.

Given this scenario, it becomes essential to invest in worker health programs that consider the specific needs of this professional category, especially in historically neglected regions. Expanding vaccination coverage, strengthening epidemiological surveillance, and implementing targeted prevention strategies can significantly contribute to reducing inequalities and improving occupational health.

Although CHWs play a fundamental role in health promotion in Brazil, it is observed that most available studies address HCW broadly, without considering their specificities^(15,33,35–39)^. This limitation indicates a gap in knowledge about the specific role of CHWs in different regional contexts.

Moreover, there is an urgent need to implement programs to encourage immunization and verify immunity, considering the disparities in vaccination coverage among the various regions of the country. Inequality in adherence to vaccination strategies can compromise the effectiveness of public health policies, making it essential to develop approaches targeted at vulnerable populations and to expand the role of CHWs in community mobilization for immunization^(50)^.

It is important to emphasize that nurses play an essential role in the health of CHWs, contributing to efficient management, health education, safe administration of vaccines, and epidemiological monitoring^(51)^. The work of these professionals is fundamental to ensuring high vaccination coverage, preventing disease outbreaks, and strengthening public health^(51)^.

The working relationship between nurses and CHWs is essential for the effectiveness of Primary Health Care actions, ensuring the implementation of the guidelines of the Brazilian Public Health System (SUS)^(52,53)^. This partnership is based on supervision, coordination, and collaboration, ensuring that the activities carried out by the CHWs are aligned with the needs of the population and public health policies^(53)^.

The nurse plays a strategic role in the efficient management of services, promoting the continuing education of community health workers and ensuring the epidemiological monitoring of working conditions. Furthermore, they are responsible for implementing biosecurity measures, including immunization, which protects both professionals and the community served^(52)^.

The integrated work between nurses and CHWs strengthens the reach of primary care, expanding access to health services and contributing to disease prevention and health promotion. Therefore, this work relationship is a fundamental pillar for improving health indicators and for equity in care for the population^(52)^.

Some limitations should be considered in this study. Due to the diversity of nomenclatures for CHWs, it is possible that some studies were not included in the search, although we have tried to minimize this limitation by adding additional keywords. Moreover, it is worth noting that this type of review does not aim to assess the methodological quality of the studies.

## CONCLUSION

The information gathered through this scoping review could not estimate the overall prevalence of serological markers or vaccination among CHWs due to a lack of scientific evidence and deparameterization of outcome measures. A research agenda is required to address the knowledge gap regarding occupational exposure to the hepatitis B virus among CHWs, with topics related to the main occupational exposures in different countries/spheres of activity, studies to identify the prevalence of serological markers and vaccination, and biosafety training in the curriculum for these professionals.

## DATA AVAILABILITY

The entire dataset supporting the results of this study is available upon request to the corresponding author.
