# Supplementary material for: Seroprevalence of markers and immunization for hepatitis B in community health workers: a scoping review
Source: Rev Esc Enferm USP. 2026 Mar 9;60:e20250358. doi: 10.1590/1980-220X-REEUSP-2025-0358en (PMC12991411; doi:10.1590/1980-220X-REEUSP-2025-0358en)
Supplement: Translation

# Translation: Soroprevalência dos marcadores e imunização para hepatite B em agentes comunitários de saúde: revisão de escopo

**DOI:** 10.1590/1980-220X-REEUSP-2025-0358pt

**Authors:** Tauana de Souza Amaral, Sara Oliveira Souza, Márcia Alves Dias de Matos, Gláucia Oliveira Abreu Batista Meireles, Cácia Régia de Paula, Marcos André de Matos

**Affiliations:** 1 Universidade Federal de Goiás, Programa de Pós-Graduação em Enfermagem e Saúde, Goiânia, GO, Brasil.; 2 Universidade Federal de Goiás, Instituto de Patologia Tropical e Saúde Pública, Programa de Pós-Graduação em Biologia das Relações Parasito-Hospedeiro, Goiânia, GO, Brasil.; 3 Associação Educativa Unievangélica, Anápolis, GO, Brasil.; 4 Universidade Federal de Jataí, Jataí, GO, Brasil.

**Keywords:** Agentes Comunitários de Saúde, Hepatite B, Biomarcadores, Vacinação, Atenção Primária à Saúde

## Abstract

**Objetivo:**

Mapear as evidências científicas acerca da prevalência do vírus da hepatite B (VHB) e da imunização entre Agentes Comunitários de Saúde (ACS) mundialmente.

**Método:**

Revisão de escopo realizada nas bases de dados LILACS, Medline (via PubMed), Embase e Scopus, além da literatura cinzenta (Google Scholar, Pro-Quest), empregando as recomendações do PRISMA-ScR (extensão para revisão de escopo) e do Manual do *Instituto Joanna Briggs*.

**Resultados:**

Foram incluídos 11 estudos, todos realizados no Brasil e sem parametrização da medida de desfecho. A prevalência estimada de infecção pelo VHB situou-se entre 8,2% e 11,1%, enquanto a cobertura vacinal apresentou variação entre 41% e 88,8%. Apenas um estudo utilizou o marcador sorológico anti-HBs para avaliar a imunização, estimando 71,6% de ACS imunizados. Dois estudos adicionais basearam-se no autorrelato do anti-HBs, com prevalências de 30,0% e 30,2%.

**Conclusão:**

A estimativa global da prevalência da infecção pelo VHB e imunização entre os ACS não pôde ser determinada em virtude da escassez e limitação geográfica dos estudos existentes, assim como a desparametrização das medidas.

## INTRODUÇÃO

A infecção pelo vírus da hepatite B (VHB) representa um desafio global, pois é uma das principais causas de doença hepática crônica, levando a aproximadamente 1,1 milhão de mortes anuais, principalmente devido à cirrose e ao câncer de fígado^(1)^. De acordo com estimativas atualizadas da Organização Mundial da Saúde (OMS), cerca de 254 milhões de pessoas vivem com hepatite B em todo o mundo, com uma carga desproporcionalmente alta na população jovem e de países de baixa e média renda^(2)^. Neste cenário, a OMS enfatiza a necessidade urgente de eliminar a hepatite B como uma ameaça à saúde pública até 2030. No entanto, os dados revelam que, até o final de 2022, apenas 13% das pessoas vivendo com infecção crônica por hepatite B haviam sido diagnosticadas e aproximadamente 3% tinham recebido terapia antiviral^(2)^.

O VHB é um dos principais agentes etiológicos de infecções ocupacionais entre os Trabalhadores da Área da Saúde (TAS), devido à exposição frequente ao sangue e outros fluidos corporais no ambiente de trabalho^(3-4)^. Estima-se que os TAS apresentam até quatro vezes mais chances de se infectar pelo VHB em comparação com a população geral^(5,6)^. Anualmente, aproximadamente 3 milhões de Trabalhadores da Área da Saúde são expostos ao vírus da hepatite B em suas atividades laborais, resultando em até 66 mil infecções pelo VHB^(6)^.

Uma vez infectados, esses trabalhadores, além de apresentarem risco de evoluir para a forma crônica da doença, podem transmitir o VHB aos pacientes sob seus cuidados. Isso reforça a importância desse grupo aderir rigorosamente às medidas de precaução, uma vez que, quando seguidas adequadamente, a transmissão é rara^(7)^. Em razão do maior risco ocupacional, os Centros de Controle e Prevenção de Doenças (CDC) e a OMS recomendam a vacinação contra o VHB como medida de profilaxia pré-exposição para todos os trabalhadores que atuam em instituições de saúde, seja os que prestam cuidados diretos ou indiretos. Ainda, a infecção pelo VHB é considerada uma das poucas Infecções Sexualmente Transmissíveis imunopreveníveis^(3,8)^.

Já há evidências robustas acerca da alta eficácia e segurança da vacina contra a hepatite B, inclusive com redução significativa nos casos ao longo dos anos com a implementação gradual de programas de imunização bem-sucedidos^(9–10)^. Estudo de avaliação global das políticas de vacinação identificou que entre os profissionais de saúde, a vacina contra a hepatite B é o imunobiológico mais amplamente adotado nas políticas de cuidados para esses trabalhadores^(11)^. Todavia, autores sublinham que o aumento do quantitativo de profissionais de saúde não imunizados e não respondedores à vacinação representa uma séria preocupação de saúde pública^(4,12)^.

Nessa perspectiva, diversos estudos caracterizam o status sorológico e imunológico dos TAS em diversos ambientes laborais, inclusive na Atenção Primária à Saúde (APS)^(3,4,8,13–14)^. No entanto, o conhecimento sobre essa temática entre os Agentes Comunitários de Saúde (ACS) a nível mundial ainda é limitado^(15–16)^, muito possivelmente decorrente da ausência de um conceito universal dessa categoria de trabalhadores, bem como clareza quanto às suas atribuições nas equipes de saúde da APS^(17)^. Embora haja incipiência nos estudos com os ACS, os identificados têm mostrado que esse grupo desempenha funções laborais que muitas vezes ultrapassam os limites das suas atribuições, expondo-os a material biológico^(16,18)^, e ainda apresenta baixo conhecimento sobre a hepatite B^(19)^. Ademais, os ACS são em sua grande maioria adultos jovens, vulneráveis a comportamentos de risco sexual^(20)^.

Diante disso, conduzimos uma revisão de escopo com o objetivo de mapear as evidências científicas sobre a prevalência do VHB e imunização entre os ACS, visando auxiliar na formulação de estratégias para garantir o cumprimento dos Objetivos de Desenvolvimento Sustentável e a Cobertura Universal de Saúde. Antes da condução desta revisão, uma pesquisa preliminar foi realizada nas bases de dados PROSPERO, Open Science Framework, Figshare, MEDLINE via PubMed, JBI Evidence Synthesis e Cochrane Database para averiguar a existência de revisões sistemáticas ou de escopo sobre o assunto; no entanto, não foi encontrado nenhum estudo ou protocolo.

## MÉTODO

### Desenho do Estudo

Trata-se de uma revisão de escopo conduzida de acordo com a metodologia do *Joanna Briggs Institute (JBI)*(21), registrada no *Open Science Framework* (https://doi.org/10.17605/OSF.IO/36YRP), a qual seguiu um protocolo a priori publicado e revisado por pares^(22)^. A extensão PRISMA para Revisões de Escopo (PRISMA-ScR) foi utilizada para o relato transparente dos dados^(23)^.

### Pergunta de Pesquisa

Qual a prevalência global do vírus da hepatite B em Agentes Comunitários de Saúde? Qual a porcentagem desses trabalhadores imunizados?

### Critérios de Elegibilidade

#### Participantes

Esta revisão considerou estudos que incluíam Agentes Comunitários de Saúde como trabalhadores que prestam cuidados de promoção, prevenção e educação em saúde ao indivíduo e família por meio das visitas domiciliares e suporte em serviços de saúde de cuidados primários^(24)^.

#### Conceito

Os marcadores sorológicos e a vacinação foram considerados na análise para identificação do status sorológico dos ACS frente ao vírus da hepatite B^(25–27)^:

- Indivíduos infectados pelo vírus da hepatite B: positividade para HBsAg e/ou anti-HBc total
- Indivíduos imunizados: anti-HBs ≥ 10 mUI/ml
- Indivíduos vacinados: relato vacinal ou verificação do cartão vacinal com três doses da vacina contra o vírus.

#### Contexto

Esta revisão considerou estudos realizados em estabelecimentos que prestam os cuidados primários em saúde, definidos como o fornecimento de ações e serviços de saúde de acordo com a necessidade da população local, baseados em evidência científica, com baixo custo e considerado o primeiro passo para o contato do indivíduo com os demais sistemas de assistência à saúde^(28)^.

#### Tipos de Fontes de Evidências

Foram incluídos como fontes de evidências artigos primários na íntegra, publicados em qualquer idioma, com desenhos de estudos quantitativos, qualitativos ou mistos. Preprints, dissertações, teses e literatura cinzenta também foram consultadas. As revisões foram excluídas do estudo. Além disso, uma busca manual nas listas de referências dos registros elegíveis para leitura do texto completo foi realizada com o objetivo de identificar artigos adicionais.

### Estratégia de Busca

Uma busca preliminar com as palavras-chave “Agentes Comunitários de Saúde” e “hepatite B” foi conduzida no MEDLINE via Pubmed para identificar se havia estudos elegíveis sobre o tópico do estudo. Após essa etapa, realizou-se a escolha das bases de dados que comporiam esta revisão. A estratégia de busca aconteceu na Literatura Latino-America e do Caribe em Ciências da Saúde (LILACS), Medline via Pubmed, Embase e Scopus. A extensão PRISMA-Statement for Reporting Literature Searches in Systematic Reviews (PRISMA-S) foi utilizada para o relato transparente da estratégia de busca (Quadro 1).

**Quadro 1:** Estratégia de busca, conduzida em 28 de janeiro de 2025 – Goiânia, GO, Brasil, 2025.

Base de dados	Estratégia de Busca	Registros Encontrados (n)
Pubmed/Medline	(“Community Health Workers” OR “Barefoot Doctor” OR “Barefoot Doctors” OR “Community Health Aide” OR “Community Health Aides” OR “Community Health Worker” OR “Family Planning Personnel” OR “Family Planning Personnel Characteristics” OR “Village Health Worker” OR “Village Health Workers” OR “health auxiliary” OR “asha” OR “accredited social health activist” OR “asha workers” OR “auxiliary health worker” OR “community health officers” OR “community health practitioner” OR “Lay health worker” OR “lay health advisor” OR “ lay health advisors” OR “lay health promoter” OR “lay health promoters” OR “lay health workers” OR “lay healthworker”) AND (“Hepatitis B” OR “Hepatitis B Virus Infection” OR “hbv infection” OR “hepatitis b infection” OR “hepatitis b viral infection” OR “hepatitis b virus infection” OR “infection by hbv” OR “infection by hepatitis b virus” OR “serum hepatitis” OR “type b hepatitis” OR “viral hepatitis b” OR “viral hepatitis type b” OR “virus hepatitis type b”)	53
Embase	(‘community health workers’/exp OR ‘community health workers’:ti,kw OR ‘barefoot doctor’/exp OR ‘barefoot doctor’:ti,kw OR ‘barefoot doctors’/exp OR ‘barefoot doctors’:ti,kw OR ‘community health aide’:ti,kw OR ‘community health aides’/exp OR ‘community health aides’:ti,kw OR ‘community health worker’/exp OR ‘community health worker’:ti,kw OR ‘family planning personnel’/exp OR ‘family planning personnel’:ti,kw OR ‘family planning personnel characteristics’/exp OR ‘family planning personnel characteristics’:ti,kw OR ‘village health worker’/exp OR ‘village health worker’:ti,kw OR ‘village health workers’:ti,kw OR ‘health auxiliary’/exp OR ‘health auxiliary’:ti,kw OR ‘asha’:ti,kw OR ‘accredited social health activist’/exp OR ‘accredited social health activist’:ti,kw OR ‘asha workers’/exp OR ‘asha workers’:ti,kw OR ‘auxiliary health worker’/exp OR ‘auxiliary health worker’:ti,kw OR ‘community health officers’/exp OR ‘community health officers’:ti,kw OR ‘community health practitioner’/exp OR ‘community health practitioner’:ti,kw OR ‘lay health worker’/exp OR ‘lay health worker’:ti,kw OR ‘lay health advisor’/exp OR ‘lay health advisor’:ti,kw OR ‘lay health advisors’/exp OR ‘lay health advisors’:ti,kw OR ‘lay health promoter’/exp OR ‘lay health promoter’:ti,kw OR ‘lay health promoters’/exp OR ‘lay health promoters’:ti,kw OR ‘lay health workers’/exp OR ‘lay health workers’:ti,kw OR ‘lay healthworker’/exp OR ‘lay healthworker’:ti,kw) AND (‘hepatitis b’/mj OR ‘hbv infection’/mj OR ‘hepatitis b infection’/mj OR ‘hepatitis b viral infection’/mj OR ‘hepatitis b virus infection’/mj OR ‘infection by hbv’/mj OR ‘infection by hepatitis b virus’/mj OR ‘serum hepatitis’/mj OR ‘type b hepatitis’/mj OR ‘viral hepatitis b’/mj OR ‘viral hepatitis type b’/mj OR ‘virus hepatitis type b’/mj)	21
BVS/Lilacs	(“Community Health Workers” OR “Barefoot Doctor” OR “Barefoot Doctors” OR “Community Health Aide” OR “Community Health Aides” OR “Community Health Worker” OR “Family Planning Personnel” OR “Family Planning Personnel Characteristics” OR “Village Health Worker” OR “Village Health Workers” OR “health auxiliary” OR “asha” OR “accredited social health activist” OR “asha workers” OR “auxiliary health worker” OR “community health officers” OR “community health practitioner” OR “Lay health worker” OR “lay health advisor” OR “ lay health advisors” OR “lay health promoter” OR “lay health promoters” OR “lay health workers” OR “lay healthworker”) AND (“Hepatitis B” OR “Hepatitis B Virus Infection” OR “hbv infection” OR “hepatitis b infection” OR “hepatitis b viral infection” OR “hepatitis b virus infection” OR “infection by hbv” OR “infection by hepatitis b virus” OR “serum hepatitis” OR “type b hepatitis” OR “viral hepatitis b” OR “viral hepatitis type b” OR “virus hepatitis type b”)	2
Web of Science	(TS = (“Community Health Workers” OR “Barefoot Doctor” OR “Barefoot Doctors” OR “Community Health Aide” OR “Community Health Aides” OR “Community Health Worker” OR “Family Planning Personnel” OR “Family Planning Personnel Characteristics” OR “Village Health Worker” OR “Village Health Workers” OR “health auxiliary” OR “asha” OR “accredited social health activist” OR “asha workers” OR “auxiliary health worker” OR “community health officers” OR “community health practitioner” OR “Lay health worker” OR “lay health advisor” OR “ lay health advisors” OR “lay health promoter” OR “lay health promoters” OR “lay health workers” OR “lay healthworker”)) AND TS=(“Hepatitis B” OR “Hepatitis B Virus Infection” OR “hbv infection” OR “hepatitis b infection” OR “hepatitis b viral infection” OR “hepatitis b virus infection” OR “infection by hbv” OR “infection by hepatitis b virus” OR “serum hepatitis” OR “type b hepatitis” OR “viral hepatitis b” OR “viral hepatitis type b” OR “virus hepatitis type b”)	41

Uma busca adicional foi realizada na base MedRxiv para a busca de preprints, e no Google Acadêmico, OpenGrey, ProQuest Dissertations and Theses Global, Catálogo de Teses e Dissertações via portal Periódicos Capes e Biblioteca Digital Brasileira de Teses e Dissertações (BDTD) para a consulta da literatura cinzenta. Foram consideradas apenas as 200 primeiras publicações relevantes disponíveis no Google Acadêmico^(29)^.

A busca foi realizada por meio dos seguintes descritores em língua inglesa, de acordo com o acrônimo PCC (Participantes: Community Health Workers, Conceito: Hepatitis B, Contexto: Primary Health Care). Como houve diferentes conceitos de ACS na literatura, algumas palavras-chave (Quadro 1) foram utilizadas para ampliar a busca^(17,30,31)^. Ademais, devido à ausência de artigos identificados na busca preliminar utilizando o acrônimo do contexto (Atenção Primária), este não foi incluído na busca final. Não houve restrição de idioma ou ano para a seleção dos artigos.

### Triagem e Seleção das Fontes de Evidências

As citações identificadas foram combinadas e carregadas no *EndNote Online* (Clarivate Analytics, PA, USA) para a remoção das duplicatas. Um teste piloto usando seis referências foi realizado previamente por dois revisores para a concordância da elegibilidade dos estudos. Em seguida, os estudos foram inseridos no software Rayyan para a triagem em duas etapas. Na etapa 1, dois revisores (TSA e SOS), independentemente, leram os títulos e resumos e selecionaram aqueles que possivelmente atendessem aos critérios de elegibilidade. Já na etapa 2, o texto completo foi examinado. Os dois revisores fizeram uma reunião de consenso em cada etapa e quaisquer divergências que surgiram na seleção dos registros foram resolvidas por meio de discussão ou com um terceiro revisor (MAM). Os resultados da pesquisa bibliográfica e do processo de inclusão dos estudos foram relatados integralmente na revisão de escopo final, e um fluxograma foi incluído para apresentar as etapas de seleção dos estudos (Figura 1).

**Figura 1:** Fluxograma de busca, seleção e inclusão das fontes de evidências – Goiânia, GO, Brasil, 2025.

### Extração de Dados

A extração de dados foi realizada pelo pesquisador TSA e o revisor SOS verificou os dados descritos. Um instrumento contendo informações sobre a publicação, características do estudo, participantes do estudo e resultados foi elaborado para essa fase, conforme as sugestões do JBI (Quadro 2). Previamente, um teste piloto foi conduzido pelos revisores para verificação da adequabilidade do instrumento.

**Quadro 2:** Síntese dos estudos incluídos na revisão - Goiânia, GO, Brasil, 2025.

Citação (autor e ano)	Estado/País	Objetivo do estudo	Tipo de estudo e período de coleta de dados	População (n)	Amostra TAS (n)	Amostra ACS (n)	Testes/estratégias	Marcadores sorológicos	Prevalência HBV	Prevalência vacinação	Prevalência imunização	Fatores associados
Sanches, 2007^(15)^	Mato Grosso do Sul/Brasil	Avaliar o status imune dos profissionais de saúde da Atenção Básica em relação a hepatite B, bem como delinear os fatores epidemiológicos que compõem o quadro representativo dessa enfermidade nesses profissionais.	Transversal analítico, realizado entre setembro de 2005 a dezembro de 2006	Não especifica	347 trabalhadores da saúde, porém por motivos operacionais, os autores colheram 332 amostras de sangue	59	Kits comerciais da marca ABBOTT com a metodologia de ensaio imunoenzimático de micropartículas	HBsAg HBeAg anti-HBc anti-HBs anti-HBe	13,5% (IC95% = 4,78%–22,22%)	Não aborda	Não aborda	Não aborda
García y Facchini, 2008^(35)^	Santa Catarina/Brasil	Verificar a prevalência da vacinação completa para hepatite B, estimular a prevalência da confirmação da imunidade e investigar os fatores associados à realização do esquema vacinal completo entre os trabalhadores de unidades de saúde do município de Florianópolis, Santa Catarina, Brasil.	Transversal analítico, realizado entre outubro e dezembro de 2006	1332	1249	Não especifica	Autorrelato vacinal	Sem marcador	Não aborda	55%	Não aborda	Não aborda
Assunção et al., 2012^(36)^	Minas Gerais/Brasil	Identificar fatores associados à vacinação contra hepatite B em trabalhadores de saúde do setor municipal	Transversal analítico, realizado em 2009.	1808	1770	Não especifica	Autorrelato vacinal	Sem marcador	Não aborda	84,1%	Não aborda	Não aborda
Costa et al., 2013^(37)^	Minas Gerais/Brasil	Investigar a prevalência e os fatores associados à vacinação contra hepatite B entre trabalhadores da Atenção Primária à Saúde, e analisar os fatores considerados importantes na determinação da vacinação contra hepatite B	Transversal analítico, realizado em 2009	797	761	480	Autorrelato vacinal	Sem marcador	Não aborda	41% (IC95% = 36,59%–45,41%)	Não aborda	Não aborda
Souza et al., 2015^(38)^	Bahia/Brasil	Avaliar a prevalência e os fatores associados à vacinação para hepatite B entre trabalhadores da Atenção Primária e da Média Complexidade do setor saúde de um município da Bahia	Transversal analítico realizado no ano de 2011	707	506	Não especifica	Autorrelato vacinal	Sem marcador	Não aborda	65,3%	Não aborda	Não aborda
Rezende, 2016^(32)^	Goiás/Brasil	Analisar o risco biológico na prática laboral dos agentes comunitários de saúde	Transversal descritivo, realizado entre setembro e novembro de 2015	89	80	80	Cartão de vacina e Autorrelato vacinal	Sem marcador	Não aborda	82,5% (IC= 74,17–90,83%), porém 52,5% tinham as três doses no cartão de vacina, e 30% relataram a completude do esquema.	30,0% (IC95% = 19,96%–40,04%) por meio do relato da realização do anti-HBs	Não aborda
Souza e Araújo, 2016^(39)^	Bahia/Brasil	Investigar a situação vacinal para as quatro vacinas garantidas pelo Programa Nacional de Imunização, avaliando a prevalência de vacinação entre as categorias ocupacionais e a completude de esquema vacinal entre trabalhadores da atenção primária e média complexidade em municípios da Bahia	Transversal descritivo, realizado entre 2010 e 2012	6.191	3.084	Não especifica	Autorrelato vacinal	Sem marcador	Não aborda	88,8%	Não aborda	Não aborda
Costa el al., 2017^(33)^	Minas Gerais/Brasil	Investigar a prevalência de verificação da imunização pós-vacinação contra hepatite B entre trabalhadores da atenção primária à saúde e sua associação com fatores ocupacionais, condições gerais de saúde e medidas de autocuidado	Transversal analítico, dados coletados entre agosto e dezembro de 2011.	797	400	197	Autorrelato vacinal e de realização do anti-HBs	Sem marcador	Não aborda	Não especifica	30,2% (IC95% = 23,79%– 36,61%), por meio do relato da realização do anti-HBs.	Não aborda
Rocha, 2017^(40)^	Minas Gerais/Brasil	Analisar a situação vacinal dos Agentes Comunitários de Saúde das unidades de Atenção Primária à Saúde de um município da zona da mata mineira.	Transversal analítico, coleta realizada entre os meses de julho a outubro de 2015; e outubro de 2016 a fevereiro de 2017.	500	400	400	Autorrelato vacinal	Sem marcador	Não aborda	87,8% (IC95% = 84,59%–91,01%)	Não aborda	Tempo de trabalho do ACS foi associado com a vacina da hepatite B
Pinto et al., 2020^(41)^	Espírito Santo/Brasil	Identificar a adesão dos ACS para vacinação contra hepatite B, determinar as causas de não adesão à vacinação contra hepatite B, investigar se a prevalência de vacinação é diferente entre as cidades pesquisadas.	Transversal analítico, realizado entre abril de 2016 a abril de 2017.	664	516	516	Autorrelato vacinal	Sem marcador	Não aborda	59,7% (IC95% = 55,48%–63,92%)	Não aborda	Mais de cinco anos de experiência na profissão foi associado a adesão à vacinação contra hepatite B
Amaral et al., 2023^(16)^	Goiás/Brasil	Identificar a situação vacinal e sorológica contra hepatite B entre Agentes Comunitários de Saúde, vacinar contra o vírus da hepatite B e avaliar a resposta imunológica dos ACS suscetíveis.	Transversal descritivo realizado entre julho a dezembro de 2019, seguido de uma coorte realizada de janeiro a maio de 2020.	172	172	109	Cartão de vacina e exames sorológicos. Para a detecção do HBsAg foi utilizado teste rápido da marca Bioclin, Brasil; e para os demais marcadores anti-HBc e anti-HBs pelo método de quimiluminescência (Architec i1000tm, abbott diagnostics)	HBsAg anti-HBc anti-HBs	8,2 % (IC95% = 4,4%–15,5%)	68,8% (IC95% = 59,6%–76,7%)	71,6% (IC95% = 62,5%–79,2%	Não aborda

### Análise e Apresentação dos Resultados

As evidências incluídas foram apresentadas em um quadro síntese que contém um resumo descritivo das características dos estudos, conforme formulário de extração de dados constante no protocolo de revisão^(22)^. Devido à escassez de estudos, dois artigos foram incluídos na presente revisão por sua importância epidemiológica, apesar de não terem utilizado a titulação dos marcadores para a hepatite B, mas sim o autorrelato^(32–33)^. Outras formas de apresentação visual de dados também foram exploradas após a extração e análise de dados terem sido concluídas, como o mapa de calor e gráfico de waffle^(34)^.

## RESULTADOS

A Figura 1 representa os processos de seleção, triagem e inclusão dos registros que compuseram a revisão. Os onze estudos incluídos foram realizados entre os anos 2007 e 2023 e conduzidos somente no Brasil. Um total de 9 artigos teve como delineamento estudo de corte transversal descritivo^(15–16,32,33,35–39)^, e somente 2 artigos de corte transversal analítico, sendo verificado que em ambos o tempo de profissão estava estatisticamente associado à vacinação contra a hepatite B^(40–41)^.

Na Figura 2, encontra-se o mapa de calor do número de estudos incluídos por estado brasileiro. Observou-se que dos 11 artigos, cinco foram realizados na região Sudeste^(33,36,37,40,41)^, três no Centro-Oeste^(15,16,32)^, dois no Nordeste^(38,39)^ e um no Sul do País^(35)^.

**Figura 2:** Mapa de calor do número de estudos incluídos por estado brasileiro – Goiânia, GO, Brasil, 2025.

Verificou-se que apenas dois estudos utilizaram marcadores sorológicos da hepatite B por meio de teste sanguíneo^(15,16)^, sendo um realizado somente com os ACS^(16)^, no qual estimou-se uma prevalência global para o HBV de 8,2% (IC95%; 4,4%–15,5%)^(16)^. Todavia, para detecção do HBsAg utilizou-se o teste de imunocromatografia de fluxo lateral. Na mesma investigação também se realizou uma coorte para verificar a resposta anamnéstica do anti-HBs quantitativo^(16)^. O segundo artigo foi conduzido com todos os trabalhadores da AP e estimou uma prevalência global de hepatite B de 11,1% (IC95%= 7,72%–14,48%) e de 13,5% (IC95%= 4,78%–22,22%) considerando somente os ACS^(15)^. Neste estudo não foi avaliada a imunização de forma isolada dos ACS, mas sim de toda a amostra^(15)^.

Do total de artigos selecionados, nove^(16,32,35–41)^ abordaram a vacinação entre os ACS, com prevalências variando de 41,0% em estudo realizado na cidade de Montes Claros, Minas Gerais^(37)^ a 88,8% realizado em cinco cidades – Feira de Santana, Itabuna, Jequié, Santo Antônio de Jesus e o Distrito Sanitário do Centro Histórico em Salvador-da Bahia^(39)^ (Figura 3). Apenas dois artigos utilizaram o cartão de vacina para conferência das doses do esquema vacinal^(16,32)^. Os demais utilizaram o autorrelato^(35–41)^.

**Figura 3:** Gráfico de waffle da prevalência vacinal dos Agentes Comunitários de Saúde encontrada nos estudos. A cor roxa representa a porcentagem de ACS vacinados, enquanto a cor azul representa os não vacinados – Goiânia, GO, Brasil, 2025.

Quanto à metodologia utilizada para identificação da imunização, somente um artigo utilizou o marcador sorológico para hepatite B (anti-HBs), estimando uma prevalência de 71,6% (IC95%;62,5%–79,2%) de ACS imunizados^(16)^. Ainda, dois artigos utilizaram o autorrelato da realização desse marcador, com prevalências estimadas de 30,0%^(32)^ e 30,2%^(33)^.

## DISCUSSÃO

A análise das evidências disponíveis, conforme delineado nesta revisão de escopo, evidenciou uma escassez de estudos que investiguem de forma abrangente a prevalência global e as taxas de imunização contra a hepatite B. Apenas dois trabalhos incluídos na amostra contemplaram a avaliação dos marcadores sorológicos da hepatite B em agentes comunitários de saúde^(15,16)^. No entanto, ambos carecem de metodologias baseadas em testes laboratoriais sensíveis para a detecção precisa desses marcadores. Especificamente, o estudo de Amaral et al.^(16)^ empregou teste rápido para identificação do antígeno de superfície da hepatite B (HBsAg); contudo, a utilização exclusiva desse método pode limitar a acurácia diagnóstica, dada a possibilidade de resultados falsos-positivos. A adoção de técnicas confirmatórias, como ensaios imunoenzimáticos ou testes moleculares, seria recomendável para assegurar maior confiabilidade nos achados. A literatura científica recomenda que estudos de prevalência e cobertura vacinal incluam a avaliação dos marcadores HBsAg, anti-HBc e anti-HBs, utilizando testes mais sensíveis, de forma a garantir uma avaliação mais precisa da imunidade populacional e da circulação do vírus^(6,27)^.

Este achado evidencia a desparametrização e o desconhecimento em relação ao diagnóstico da hepatite B e serve como um importante alerta para reforçar a urgência na formação dos profissionais de saúde, qualificação profissional e da disponibilização de ferramentas laboratoriais mais sensíveis^(42)^. A prática clínica baseada em evidências enfatiza a necessidade de utilizar um painel completo dos marcadores sorológicos, uma vez que a ausência de uma testagem completa pode gerar resultados falsos positivos ou falsos negativos, tratamentos desnecessários e subestimação da imunidade populacional, dificultando a identificação de indivíduos suscetíveis e a implementação de medidas preventivas eficazes, o que pode levar a falhas na vigilância epidemiológica^(26)^.

Outrossim, nove dos artigos analisados avaliaram a soroprevalência e a imunização contra a hepatite B utilizando o autorrelato como método de coleta de dados^(32,33)^. Apesar de ser uma ferramenta prática, de baixo custo e de fácil aplicação, especialmente em estudos populacionais e em contextos onde o acesso a registros vacinais é restrito, o autorrelato apresenta limitações importantes, como o viés de memória, que pode comprometer a confiabilidade dos resultados^(6)^. Uma possível justificativa para a adoção dessa abordagem é a limitação de financiamento para pesquisas, uma vez que a realização de exames laboratoriais, embora mais precisa, envolve custos significativamente mais elevados quando comparada ao uso do autorrelato.

Estudos disponíveis indicam que a complementação do autorrelato com a análise de registros vacinais, como o cartão de vacinas, ou com testes laboratoriais, como a dosagem dos marcadores sorológicos, aumenta significativamente a precisão na avaliação da situação vacinal^(3,43)^. No entanto, o acesso ao cartão de vacinas ainda representa um obstáculo para os profissionais de saúde e pesquisadores, já que a população, em geral, não tem o hábito de considerar este documento como algo essencial que deve ser devidamente armazenado^(44)^. Nesse contexto, torna-se evidente a necessidade de informatizar o registro vacinal, embora essa realidade ainda esteja distante em muitos cenários devido às desigualdades estruturais e digitais enfrentadas pelas unidades de saúde em nível global.

Todos os estudos selecionados foram realizados no Brasil, muito possivelmente em decorrência dos ACS serem considerados profissionais de saúde^(45)^, o que demonstra a importância do Sistema Único de Saúde brasileiro. Em contraste, em outros países, os ACS frequentemente atuam como voluntários, recebem baixa remuneração e não têm o devido reconhecimento profissional^(46)^.

Além disso, esse entendimento é respaldado pela legislação específica da categoria, que estabelece diretrizes fundamentais relacionadas às condições de insalubridade, à realização de exames periódicos ocupacionais e à disponibilização e utilização adequada de Equipamentos de Proteção Individual (EPIs)^(47)^. Adicionalmente, os achados reforçam os esforços contínuos do Programa Nacional de Imunizações (PNI) do Brasil, que desempenha um papel crucial no monitoramento e na melhoria das taxas de imunização, além de contribuir para a identificação de lacunas nos sistemas de saúde locais^(48)^.

A liderança do Brasil na implementação de estratégias de vacinação e saúde pública evidencia a necessidade de ampliar pesquisas internacionais, permitindo uma comparação mais abrangente entre diferentes contextos e soluções globais.

A maioria dos estudos sobre hepatite B no Brasil foi conduzida na região Sudeste^(33,36,37,40,41)^, enquanto estados com maior prevalência da infecção, como os da região Nordeste^(38,39)^, foram sub-representados na literatura científica^(49)^. Essa disparidade evidencia as iniquidades no acesso aos cuidados em saúde, refletindo desigualdades estruturais que impactam diretamente a vigilância epidemiológica e a implementação de políticas públicas eficazes.

Diante desse cenário, torna-se essencial o investimento em programas de saúde do trabalhador que considerem as especificidades dessa categoria profissional, especialmente em regiões historicamente negligenciadas. A ampliação da cobertura vacinal, o fortalecimento da vigilância epidemiológica e a implementação de estratégias de prevenção direcionadas podem contribuir significativamente para a redução das desigualdades e para a melhoria da saúde ocupacional.

Embora os ACS desempenhem um papel fundamental na promoção da saúde no Brasil, observa-se que a maioria dos estudos disponíveis aborda os TAS de forma ampla, sem considerar as especificidades desses trabalhadores^(15,33,35–39)^. Essa limitação evidencia uma lacuna no conhecimento sobre a atuação específica dos ACS em diferentes contextos regionais.

Além disso, há uma necessidade urgente de implementação de programas de incentivo à imunização e à verificação da imunidade, considerando as disparidades nas coberturas vacinais entre as diversas regiões do país. A desigualdade na adesão às estratégias de vacinação pode comprometer a efetividade das políticas de saúde pública, tornando essencial o desenvolvimento de abordagens direcionadas para populações vulneráveis e a ampliação do papel dos ACS na mobilização comunitária para a imunização^(50)^.

Cabe destacar que o enfermeiro desempenha um papel essencial na saúde dos ACS, contribuindo para a gestão eficiente, educação em saúde, administração segura das vacinas e monitoramento epidemiológico^(51)^. A atuação desses profissionais é fundamental para garantir altas coberturas vacinais, prevenir surtos de doenças e fortalecer a saúde pública^(51)^.

A relação de trabalho entre o enfermeiro e o Agente Comunitário de Saúde é essencial para a efetividade das ações de Atenção Primária à Saúde, garantindo a implementação das diretrizes do Sistema Único de Saúde^(52,53)^. Essa parceria fundamenta-se na supervisão, coordenação e colaboração, assegurando que as atividades desenvolvidas pelos ACS estejam alinhadas às necessidades da população e às políticas públicas de saúde^(53)^.

O enfermeiro desempenha um papel estratégico na gestão eficiente dos serviços, promovendo a educação permanente dos ACS e garantindo o monitoramento epidemiológico das condições de trabalho. Além disso, é responsável pela implementação de medidas de biossegurança, incluindo a imunização, que protege tanto os profissionais quanto a comunidade atendida^(52)^.

A atuação integrada entre enfermeiros e ACS fortalece a capilaridade da atenção primária, ampliando o acesso aos serviços de saúde e contribuindo para a prevenção de doenças e promoção da saúde. Dessa forma, essa relação de trabalho é um pilar fundamental para a melhoria dos indicadores de saúde e para a equidade no atendimento à população^(52)^.

Algumas limitações devem ser consideradas nesse estudo. Devido à diversidade de nomenclaturas para os ACS, é possível que alguns estudos não tenham sido incluídos na busca, embora tenhamos tentado minimizar essa limitação por meio da adição de palavras-chave adicionais. Além disso, vale ressaltar que este tipo de revisão não tem como objetivo avaliar a qualidade metodológica dos estudos.

## CONCLUSÃO

As informações levantadas por meio dessa revisão de escopo não puderam estimar a prevalência global dos marcadores sorológicos nem da vacinação entre os ACS, devido à falta de evidências científicas e desparametrização das medidas de desfecho. Uma agenda de pesquisa é necessária para sanar a lacuna de conhecimento sobre a exposição ocupacional ao vírus da hepatite B entre os ACS, com temas relacionados às principais exposições ocupacionais em diferentes países/esferas de atuação, estudos para a identificação da prevalência dos marcadores sorológicos e da vacinação, e a formação em biossegurança na curricularização desses profissionais.

## DISPONIBILIDADE DE DADOS

Todo o conjunto de dados que dá suporte aos resultados deste estudo está disponível mediante solicitação ao autor correspondente.

# Translation: Seroprevalencia de marcadores e inmunización contra la hepatitis B en agentes comunitarios de salud: revisión de alcance

**DOI:** 10.1590/1980-220X-REEUSP-2025-0358es

**Authors:** Tauana de Souza Amaral, Sara Oliveira Souza, Márcia Alves Dias de Matos, Gláucia Oliveira Abreu Batista Meireles, Cácia Régia de Paula, Marcos André de Matos

**Affiliations:** 1 Universidade Federal de Goiás, Programa de Pós-Graduação em Enfermagem e Saúde, Goiânia, GO, Brasil.; 2 Universidade Federal de Goiás, Instituto de Patologia Tropical e Saúde Pública, Programa de Pós-Graduação em Biologia das Relações Parasito-Hospedeiro, Goiânia, GO, Brasil.; 3 Associação Educativa Unievangélica, Anápolis, GO, Brasil.; 4 Universidade Federal de Jataí, Jataí, GO, Brasil.

**Keywords:** Agentes Comunitarios de Salud, Hepatitis B, Biomarcadores, Vacunación, Atención Primaria de Salud

## Abstract

**Objetivo:**

Mapear la evidencia científica relativa a la prevalencia del virus de la hepatitis B (VHB) y la inmunización entre los trabajadores de salud comunitarios (TSC) en todo el mundo.

**Método:**

Se realizó una revisión del alcance utilizando las bases de datos LILACS, Medline (a través de PubMed), Embase y Scopus, así como literatura gris (Google Scholar, ProQuest), empleando las recomendaciones de PRISMA-ScR (extensión para revisiones exploratorias) y el Manual del *Instituto Joanna Briggs*.

**Resultados:**

Se incluyeron once estudios, todos realizados en Brasil y sin parametrización de la medida de resultado. La prevalencia estimada de la infección por VHB osciló entre el 8,2% y el 11,1%, mientras que la cobertura de vacunación varió entre el 41% y el 88,8%. Solo un estudio utilizó el marcador serológico anti-HBs para evaluar la inmunización, estimando que el 71,6% de los TSC estaban inmunizados. Dos estudios adicionales se basaron en autoinformes de anti-HBs, con prevalencias del 30,0% y el 30,2%.

**Conclusión:**

No se pudo determinar la estimación global de la prevalencia de la infección por VHB y la inmunización entre los TSC debido a la escasez y las limitaciones geográficas de los estudios existentes, así como a la falta de mediciones estandarizadas.

## INTRODUCCIÓN

La infección por el virus de la hepatitis B (VHB) representa un desafío global, ya que es una de las principales causas de enfermedad hepática crónica, lo que provoca aproximadamente 1,1 millones de muertes al año, principalmente debido a la cirrosis y al cáncer de hígado^(1)^. Según las estimaciones actualizadas de la Organización Mundial de la Salud (OMS), alrededor de 254 millones de personas viven con hepatitis B en todo el mundo, con una carga desproporcionadamente alta en la población joven y en los países de ingresos bajos y medios^(2)^. En este contexto, la OMS hace hincapié en la urgente necesidad de eliminar la hepatitis B como amenaza para la salud pública para 2030. Sin embargo, los datos revelan que, a finales de 2022, solo el 13 % de las personas que vivían con infección crónica por hepatitis B habían sido diagnosticadas y aproximadamente el 3 % había recibido terapia antiviral^(2)^.

El VHB es uno de los principales agentes etiológicos de las infecciones ocupacionales entre los trabajadores de la salud (TAS), debido a la exposición frecuente a la sangre y otros fluidos corporales en el entorno laboral^(3,4)^. Se estima que los TAS tienen hasta cuatro veces más probabilidades de infectarse por el VHB en comparación con la población general^(5,6)^. Cada año, aproximadamente 3 millones de trabajadores del sector sanitario se exponen al virus de la hepatitis B en sus actividades laborales, lo que da lugar a hasta 66 000 infecciones por el VHB^(6)^.

Una vez infectados, estos trabajadores, además de correr el riesgo de desarrollar la forma crónica de la enfermedad, pueden transmitir el VHB a los pacientes bajo su cuidado. Esto refuerza la importancia de que este grupo cumpla rigurosamente las medidas de precaución, ya que, cuando se siguen adecuadamente, la transmisión es poco frecuente^(7)^. Debido al mayor riesgo ocupacional, los Centros para el Control y la Prevención de Enfermedades (CDC) y la OMS recomiendan la vacunación contra el VHB como medida de profilaxis previa a la exposición para todos los trabajadores que actúan en instituciones de salud, ya sea que presten cuidados directos o indirectos. Además, la infección por el VHB se considera una de las pocas infecciones de transmisión sexual prevenibles mediante inmunización^(3,8)^.

Ya existen pruebas sólidas sobre la alta eficacia y seguridad de la vacuna contra la hepatitis B, incluida una reducción significativa de los casos a lo largo de los años con la implementación gradual de programas de inmunización exitosos^(9,10)^. Un estudio de evaluación global de las políticas de vacunación identificó que, entre los profesionales de la salud, la vacuna contra la hepatitis B es el inmunobiológico más ampliamente adoptado en las políticas de atención a estos trabajadores^(11)^. Sin embargo, los autores subrayan que el aumento del número de profesionales de la salud no inmunizados y que no responden a la vacunación representa una grave preocupación para la salud pública^(4,12)^.

Desde esta perspectiva, varios estudios caracterizan el estado serológico e inmunológico de los TAS en diversos entornos laborales, incluida la Atención Primaria de Salud (APS)^(3,4,8,13,14)^. Sin embargo, el conocimiento sobre este tema entre los Agentes Comunitarios de Salud (ACS) a nivel mundial sigue siendo limitado^(15,16)^, muy posiblemente debido a la ausencia de un concepto universal de esta categoría de trabajadores, así como a la falta de claridad sobre sus funciones en los equipos de salud de la APS^(17)^. Aunque los estudios con ACS son incipientes, los identificados han demostrado que este grupo desempeña funciones laborales que a menudo exceden los límites de sus atribuciones, exponiéndolos a material biológico^(16,18)^, y aún presenta un bajo conocimiento sobre la hepatitis B^(19)^. Además, la mayoría de los ACS son adultos jóvenes, vulnerables a comportamientos sexuales de riesgo^(20)^.

Ante esto, realizamos una revisión de alcance con el objetivo de mapear la evidencia científica sobre la prevalencia del VHB y la inmunización entre los ACS, con el fin de ayudar en la formulación de estrategias para garantizar el cumplimiento de los Objetivos de Desarrollo Sostenible y la Cobertura Universal de Salud. Antes de realizar esta revisión, se llevó a cabo una investigación preliminar en las bases de datos PROSPERO, Open Science Framework, Figshare, MEDLINE a través de PubMed, JBI Evidence Synthesis y Cochrane Database para averiguar la existencia de revisiones sistemáticas o de alcance sobre el tema; sin embargo, no se encontró ningún estudio o protocolo.

## MÉTODO

### Diseño del Estudio

Se trata de una revisión de alcance realizada de acuerdo con la metodología del *Joanna Briggs Institute (JBI)*(21), registrada en el *Open Science Framework* (https://doi.org/10.17605/OSF.IO/36YRP), que siguió un protocolo a priori publicado y revisado por pares^(22)^. Se utilizó la extensión PRISMA para revisiones de alcance (PRISMA-ScR) para la presentación transparente de los datos^(23)^.

### Pregunta de Investigación

¿Cuál es la prevalencia global del virus de la hepatitis B en los agentes comunitarios de salud? ¿Qué porcentaje de estos trabajadores está inmunizado?

### Criterios de Elegibilidad

#### Participantes

Esta revisión consideró estudios que incluían a los agentes comunitarios de salud como trabajadores que prestan servicios de promoción, prevención y educación en salud al individuo y a la familia mediante visitas domiciliarias y apoyo en los servicios de salud de atención primaria^(24)^.

#### Concepto

Los marcadores serológicos y la vacunación se consideraron en el análisis para identificar el estado serológico de los ACS frente al virus de la hepatitis B^(25–27)^:

- Individuos infectados por el virus de la hepatitis B: positividad para HBsAg y/o anti-HBc total
- Individuos inmunizados: anti-HBs ≥ 10 mUI/ml
- Individuos vacunados: informe de vacunación o verificación de la cartilla de vacunación con tres dosis de la vacuna contra el virus.

#### Contexto

Esta revisión consideró estudios realizados en establecimientos que prestan atención primaria en salud, definida como la prestación de acciones y servicios de salud de acuerdo con las necesidades de la población local, basados en evidencia científica, de bajo costo y considerados el primer paso para el contacto del individuo con los demás sistemas de asistencia sanitaria^(28)^.

#### Tipos de Fuentes de Evidencia

Se incluyeron como fuentes de evidencia artículos primarios completos, publicados en cualquier idioma, con diseños de estudios cuantitativos, cualitativos o mixtos. También se consultaron preimpresiones, disertaciones, tesis y literatura gris. Las revisiones se excluyeron del estudio. Además, se realizó una búsqueda manual en las listas de referencias de los registros elegibles para la lectura del texto completo con el objetivo de identificar artículos adicionales.

### Estrategia de Búsqueda

Se realizó una búsqueda preliminar con las palabras clave «Agentes Comunitarios de Salud» y «hepatitis B» en MEDLINE a través de Pubmed para identificar si había estudios elegibles sobre el tema del estudio. Tras esta etapa, se seleccionaron las bases de datos que componerían esta revisión. La estrategia de búsqueda se llevó a cabo en la Literatura Latinoamericana y del Caribe en Ciencias de la Salud (LILACS), Medline a través de Pubmed, Embase y Scopus. Se utilizó la extensión PRISMA-Statement for Reporting Literature Searches in Systematic Reviews (PRISMA-S) para informar de forma transparente sobre la estrategia de búsqueda (Cuadro 1).

**Cuadro 1:** Estrategia de búsqueda, llevada a cabo el 28 de enero de 2025 – Goiânia, GO, Brasil, 2025.

Base de datos	Estrategia de Búsqueda	Registros Encontrados (n)
Pubmed/Medline	(“Community Health Workers” OR “Barefoot Doctor” OR “Barefoot Doctors” OR “Community Health Aide” OR “Community Health Aides” OR “Community Health Worker” OR “Family Planning Personnel” OR “Family Planning Personnel Characteristics” OR “Village Health Worker” OR “Village Health Workers” OR “health auxiliary” OR “asha” OR “accredited social health activist” OR “asha workers” OR “auxiliary health worker” OR “community health officers” OR “community health practitioner” OR “Lay health worker” OR “lay health advisor” OR “ lay health advisors” OR “lay health promoter” OR “lay health promoters” OR “lay health workers” OR “lay healthworker”) AND (“Hepatitis B” OR “Hepatitis B Virus Infection” OR “hbv infection” OR “hepatitis b infection” OR “hepatitis b viral infection” OR “hepatitis b virus infection” OR “infection by hbv” OR “infection by hepatitis b virus” OR “serum hepatitis” OR “type b hepatitis” OR “viral hepatitis b” OR “viral hepatitis type b” OR “virus hepatitis type b”)	53
Embase	(‘community health workers’/exp OR ‘community health workers’:ti,kw OR ‘barefoot doctor’/exp OR ‘barefoot doctor’:ti,kw OR ‘barefoot doctors’/exp OR ‘barefoot doctors’:ti,kw OR ‘community health aide’:ti,kw OR ‘community health aides’/exp OR ‘community health aides’:ti,kw OR ‘community health worker’/exp OR ‘community health worker’:ti,kw OR ‘family planning personnel’/exp OR ‘family planning personnel’:ti,kw OR ‘family planning personnel characteristics’/exp OR ‘family planning personnel characteristics’:ti,kw OR ‘village health worker’/exp OR ‘village health worker’:ti,kw OR ‘village health workers’:ti,kw OR ‘health auxiliary’/exp OR ‘health auxiliary’:ti,kw OR ‘asha’:ti,kw OR ‘accredited social health activist’/exp OR ‘accredited social health activist’:ti,kw OR ‘asha workers’/exp OR ‘asha workers’:ti,kw OR ‘auxiliary health worker’/exp OR ‘auxiliary health worker’:ti,kw OR ‘community health officers’/exp OR ‘community health officers’:ti,kw OR ‘community health practitioner’/exp OR ‘community health practitioner’:ti,kw OR ‘lay health worker’/exp OR ‘lay health worker’:ti,kw OR ‘lay health advisor’/exp OR ‘lay health advisor’:ti,kw OR ‘lay health advisors’/exp OR ‘lay health advisors’:ti,kw OR ‘lay health promoter’/exp OR ‘lay health promoter’:ti,kw OR ‘lay health promoters’/exp OR ‘lay health promoters’:ti,kw OR ‘lay health workers’/exp OR ‘lay health workers’:ti,kw OR ‘lay healthworker’/exp OR ‘lay healthworker’:ti,kw) AND (‘hepatitis b’/mj OR ‘hbv infection’/mj OR ‘hepatitis b infection’/mj OR ‘hepatitis b viral infection’/mj OR ‘hepatitis b virus infection’/mj OR ‘infection by hbv’/mj OR ‘infection by hepatitis b virus’/mj OR ‘serum hepatitis’/mj OR ‘type b hepatitis’/mj OR ‘viral hepatitis b’/mj OR ‘viral hepatitis type b’/mj OR ‘virus hepatitis type b’/mj)	21
BVS/Lilacs	(“Community Health Workers” OR “Barefoot Doctor” OR “Barefoot Doctors” OR “Community Health Aide” OR “Community Health Aides” OR “Community Health Worker” OR “Family Planning Personnel” OR “Family Planning Personnel Characteristics” OR “Village Health Worker” OR “Village Health Workers” OR “health auxiliary” OR “asha” OR “accredited social health activist” OR “asha workers” OR “auxiliary health worker” OR “community health officers” OR “community health practitioner” OR “Lay health worker” OR “lay health advisor” OR “ lay health advisors” OR “lay health promoter” OR “lay health promoters” OR “lay health workers” OR “lay healthworker”) AND (“Hepatitis B” OR “Hepatitis B Virus Infection” OR “hbv infection” OR “hepatitis b infection” OR “hepatitis b viral infection” OR “hepatitis b virus infection” OR “infection by hbv” OR “infection by hepatitis b virus” OR “serum hepatitis” OR “type b hepatitis” OR “viral hepatitis b” OR “viral hepatitis type b” OR “virus hepatitis type b”)	2
Web of Science	(TS = (“Community Health Workers” OR “Barefoot Doctor” OR “Barefoot Doctors” OR “Community Health Aide” OR “Community Health Aides” OR “Community Health Worker” OR “Family Planning Personnel” OR “Family Planning Personnel Characteristics” OR “Village Health Worker” OR “Village Health Workers” OR “health auxiliary” OR “asha” OR “accredited social health activist” OR “asha workers” OR “auxiliary health worker” OR “community health officers” OR “community health practitioner” OR “Lay health worker” OR “lay health advisor” OR “ lay health advisors” OR “lay health promoter” OR “lay health promoters” OR “lay health workers” OR “lay healthworker”)) AND TS = (“Hepatitis B” OR “Hepatitis B Virus Infection” OR “hbv infection” OR “hepatitis b infection” OR “hepatitis b viral infection” OR “hepatitis b virus infection” OR “infection by hbv” OR “infection by hepatitis b virus” OR “serum hepatitis” OR “type b hepatitis” OR “viral hepatitis b” OR “viral hepatitis type b” OR “virus hepatitis type b”)	41

Se realizó una búsqueda adicional en la base MedRxiv para buscar preprints, y en Google Académico, OpenGrey, ProQuest Dissertations and Theses Global, Catálogo de Tesis y Disertaciones a través del portal Periódicos Capes y Biblioteca Digital Brasileña de Tesis y Disertaciones (BDTD) para consultar la literatura gris. Solo se consideraron las primeras 200 publicaciones relevantes disponibles en Google Académico^(29)^.

La búsqueda se realizó mediante los siguientes descriptores en inglés, de acuerdo con el acrónimo PCC (Participantes: Community Health Workers, Concepto: Hepatitis B, Contexto: Primary Health Care). Dado que existían diferentes conceptos de ACS en la literatura, se utilizaron algunas palabras clave (Cuadro 1) para ampliar la búsqueda^(17,30,31)^. Además, debido a la ausencia de artículos identificados en la búsqueda preliminar utilizando el acrónimo del contexto (Atención Primaria), este no se incluyó en la búsqueda final. No hubo restricciones de idioma o año para la selección de los artículos.

### Selección de las Fuentes de Evidencia

Las citas identificadas se combinaron y se cargaron en *EndNote Online* (Clarivate Analytics, PA, EE. UU.) para eliminar los duplicados. Dos revisores realizaron previamente una prueba piloto con seis referencias para acordar la elegibilidad de los estudios. A continuación, los estudios se introdujeron en el software Rayyan para su selección en dos etapas. En la etapa 1, dos revisores (TSA y SOS), de forma independiente, leyeron los títulos y resúmenes y seleccionaron aquellos que posiblemente cumplían los criterios de elegibilidad. En la etapa 2, se examinó el texto completo. Los dos revisores celebraron una reunión de consenso en cada etapa y cualquier discrepancia que surgiera en la selección de los registros se resolvió mediante discusión o con un tercer revisor (MAM). Los resultados de la búsqueda bibliográfica y del proceso de inclusión de los estudios se describieron íntegramente en la revisión del alcance final, y se incluyó un diagrama de flujo para presentar las etapas de selección de los estudios (Figura 1).

**Figura 1:** Diagrama de flujo de búsqueda, selección e inclusión de fuentes de evidencia – Goiânia, GO, Brasil, 2025.

### Extracción de Datos

La extracción de datos fue realizada por el investigador TSA y el revisor SOS verificó los datos descritos. Se elaboró un instrumento con información sobre la publicación, las características del estudio, los participantes en el estudio y los resultados para esta fase, de acuerdo con las sugerencias del JBI (Cuadro 2). Previamente, los revisores realizaron una prueba piloto para verificar la idoneidad del instrumento.

**Cuadro 2:** Resumen de los estudios incluidos en la revisión – Goiânia, GO, Brasil, 2025.

Cita (autor y año)	Estado/País	Objetivo del estudio	Tipo de estudio y período de recopilación de datos	Población (n)	Muestra TAS (n)	Muestra ACS (n)	Pruebas/estrategias	Marcadores serológicos	Prevalencia del VHB	Prevalencia de vacunación	Prevalencia de inmunización	Factores asociados
Sanches, 2007^(15)^	Mato Grosso do Sul/Brasil	Evaluar el estado inmunitario de los profesionales sanitarios de atención primaria en relación con la hepatitis B, así como delinear los factores epidemiológicos que componen el cuadro representativo de esta enfermedad en estos profesionales.	Estudio transversal analítico, realizado entre septiembre de 2005 y diciembre de 2006	No se especifica	347 trabajadores sanitarios, pero por motivos operativos, los autores recogieron 332 muestras de sangre	59	Kits comerciales de la marca ABBOTT con la metodología de ensayo inmunoenzimático de micropartículas	HBsAg HBeAg anti-HBc anti-HBs anti-HBe	13,5 % (IC95 % = 4,78 %– 22,22 %)	No aborda	No aborda	No aborda
García y Facchini, 2008^(35)^	Santa Catarina/Brasil	Verificar la prevalencia de la vacunación completa contra la hepatitis B, estimular la prevalencia de la confirmación de la inmunidad e investigar los factores asociados a la realización del esquema vacunal completo entre los trabajadores de las unidades de salud del municipio de Florianópolis, Santa Catarina, Brasil.	Estudio transversal analítico, realizado entre octubre y diciembre de 2006	1332	1249	No se especifica	Autoinforme sobre la vacunación	Sin marcador	No se aborda	55	No aborda	No aborda
Assunção et al., 2012^(36)^	Minas Gerais/Brasil	Identificar factores asociados a la vacunación contra la hepatitis B en trabajadores sanitarios del sector municipal	Estudio transversal analítico, realizado en 2009.	1808	1770	No se especifica	Autoinforme sobre vacunación	Sin marcador	No se aborda	84,1	No aborda	No se aborda
Costa et al., 2013^(37)^	Minas Gerais/Brasil	Investigar la prevalencia y los factores asociados a la vacunación contra la hepatitis B entre los trabajadores de atención primaria de salud, y analizar los factores considerados importantes en la determinación de la vacunación contra la hepatitis B.	Estudio transversal analítico, realizado en 2009	797	761	480	Autoinforme sobre vacunación	Sin marcador	No aborda	41 % (IC del 95 % = 36,59 %– 45,41 %)	No aborda	No aborda
Souza et al., 2015^(38)^	Bahía/Brasil	Evaluar la prevalencia y los factores asociados a la vacunación contra la hepatitis B entre los trabajadores de atención primaria y de complejidad media del sector sanitario de un municipio de Bahía	Estudio transversal analítico realizado en el año 2011	707	506	No se especifica	Autoinforme sobre vacunación	Sin marcador	No se aborda	65,3	No se aborda	No aborda
Rezende, 2016^(32)^	Goiás/Brasil	Analizar el riesgo biológico en la práctica laboral de los agentes comunitarios de salud	Transversal descriptivo, realizado entre septiembre y novi noviembre de 2015	89	80	80	Tarjeta de vacunación y autoinforme de vacunación	Sin marcador	No aborda	82,5 % (IC = 74,17– 90,83 %), pero el 52,5 % tenía las tres dosis en la tarjeta de vacunación, y el 30 % informó haber completado el esquema.	30,0 % (IC9 % = 19,96 %– 40,04 %) mediante el informe de la realización de la prueba anti-HBs	No se aborda
Souza y Araújo, 2016^(39)^	Bahía/Brasil	Investigar la situación de vacunación para las cuatro vacunas garantizadas por el Programa Nacional de Inmunización, evaluando la prevalencia de la vacunación entre las categorías ocupacionales y la completitud del esquema de vacunación entre los trabajadores de atención primaria y media complejidad en municipios de Bahía	Transversal descriptivo, realizado entre 2010 y 2012	6.191	3.084	No se especifica	Autoinforme sobre vacunación	Sin marcador	No se aborda	88,8	No se aborda	No aborda
Costa et al., 2017^(33)^	Minas Gerais/Brasil	Investigar la prevalencia de la verificación de la inmunización posvacunal contra la hepatitis B entre los trabajadores de atención primaria de salud y su asociación con factores ocupacionales, condiciones generales de salud y medidas de autocuidado	Transversal analítico, datos recolectados entre agosto y diciembre de 2011.	797	400	197	Autoinforme sobre la vacunación y la realización de la prueba anti-HBs	Sin marcador	No se aborda	No especifica	30,2 % (IC95 % = 23,79 %– 36,61 %), mediante el informe de la realización de la prueba anti-HBs.	No aborda
Rocha, 2017^(40)^	Minas Gerais/Brasil	Analizar la situación vacunal de los agentes comunitarios de salud de las unidades de atención primaria de salud de un municipio de la zona forestal de Minas Gerais.	Estudio transversal analítico, recopilación realizada entre los meses de julio a octubre de 2015 y octubre de 2016 a febrero de 2017.	500	400	400	Autoinforme sobre vacunación	Sin marcador	No aborda	87,8 % (IC del 95 % = 84,59 %– 91,01 %)	No aborda	El tiempo de trabajo del ACS se asoció con la vacuna contra la hepatitis B
Pinto et al., 2020^(41)^	Espírito Santo/Brasil	Identificar la adherencia de los ACS a la vacunación contra la hepatitis B, determinar las causas de la no adherencia a la vacunación contra la hepatitis B, investigar si la prevalencia de la vacunación es diferente entre las ciudades estudiadas.	Estudio transversal analítico, realizado entre abril de 2016 y abril de 2017.	664	516	516	Autoinforme sobre vacunación	Sin marcador	No aborda	59,7 % (IC del 95 % = 55,48 %– 63,92 %)	No aborda	Más de cinco años de experiencia en la profesión se asoció con la adhesión a la vacunación contra la hepatitis B
Amaral et al., 2023^(16)^	Goiás/Brasil	Identificar la situación vacunal y serológica contra la hepatitis B entre los agentes comunitarios de salud, vacunar contra el virus de la hepatitis B y evaluar la respuesta inmunológica e e de los ACS susceptibles.	Estudio transversal descriptivo realizado entre julio y diciembre de 2019, seguido de un estudio de cohorte realizado entre enero y mayo de 2020.	172	172	109	Tarjeta de vacunación y exámenes serológicos. Para la detección del HBsAg se utilizó la prueba rápida de la marca Bioclin, Brasil; y para los demás marcadores anti-HBc y anti-HBs, el método de quimioluminiscencia (Architec i1000tm, abbott diagnostics).	HBsAg anti-HBc anti-HBs	8,2 % (IC95 % = 4,4 %– 15,5 %)	68,8 % (IC95 % = 59,6 %– 76,7 %)	71,6 % (IC95 % = 62,5 %– 79,2 %	No aborda

### Análisis y Presentación de los Resultados

Las evidencias incluidas se presentaron en un cuadro resumen que contiene un resumen descriptivo de las características de los estudios, de acuerdo con el formulario de extracción de datos incluido en el protocolo de revisión^(22)^. Debido a la escasez de estudios, se incluyeron dos artículos en la presente revisión por su importancia epidemiológica, a pesar de que no utilizaron la titulación de los marcadores para la hepatitis B, sino el autoinforme^(32,33)^. También se exploraron otras formas de presentación visual de los datos una vez concluida la extracción y el análisis de los mismos, como el mapa de calor y el gráfico waffle^(34)^.

## RESULTADOS

La Figura 1 representa los procesos de selección e inclusión de los registros que componen la revisión. Los once estudios incluidos se realizaron entre los años 2007 y 2023 y se llevaron a cabo únicamente en Brasil. Un total de 9 artículos tenían un diseño transversal descriptivo^(15,16,32,33,35–39)^, y solo 2 artículos tenían un diseño transversal analítico, verificándose que en ambos el tiempo de ejercicio de la profesión estaba estadísticamente asociado a la vacunación contra la hepatitis B^(40,41)^.

En la Figura 2 se muestra el mapa de calor del número de estudios incluidos por estado brasileño. Se observó que, de los 11 artículos, cinco se realizaron en la región sudeste^(33,36,37,40,41)^, tres en la región centro-oeste^(15,16,32)^, dos en la región noreste^(38,39)^ y uno en la región sur del país^(35)^.

**Figura 2:** Mapa de calor del número de estudios incluidos por estado brasileño – Goiânia, GO, Brasil, 2025.

Se verificó que solo dos estudios utilizaron marcadores serológicos de la hepatitis B mediante análisis de sangre^(15,16)^, uno de ellos realizado únicamente con los ACS^(16)^, en el que se estimó una prevalencia global del VHB del 8,2 % (IC del 95 %; 4,4 %–15,5 %)^(16)^. Sin embargo, para la detección del HBsAg se utilizó la prueba de inmunocromatografía de flujo lateral. En la misma investigación también se realizó una cohorte para verificar la respuesta anamnésica del anti-HBs cuantitativo^(16)^. El segundo artículo se llevó a cabo con todos los trabajadores de la AP y estimó una prevalencia global de hepatitis B del 11,1 % (IC del 95 % = 7,72 %–14,48 %) y del 13,5 % (IC del 95 % = 4,78 %–22,22 %) considerando solo a los ACS^(15)^. En este estudio no se evaluó la inmunización de forma aislada de los ACS, sino de toda la muestra^(15)^.

Del total de artículos seleccionados, nueve^(16,32,35–41)^ abordaron la vacunación entre los ACS, con prevalencias que variaban entre el 41,0 % en un estudio realizado en la ciudad de Montes Claros, Minas Gerais^(37)^, y el 88,8 % en un estudio realizado en cinco ciudades —Feira de Santana, Itabuna, Jequié, Santo Antônio de Jesus y el Distrito Sanitario del Centro Histórico en Salvador, Bahía^(39)^ (Figura 3). Solo dos artículos utilizaron la cartilla de vacunación para verificar las dosis del esquema de vacunación^(16,32)^. Los demás utilizaron el autorreporte^(35–41)^.

**Figura 3:** Gráfico waffle de la prevalencia de vacunación de los agentes comunitarios de salud encontrada en los estudios. El color morado representa el porcentaje de ACS vacunados, mientras que el color azul representa a los no vacunados – Goiânia, GO, Brasil, 2025.

En cuanto a la metodología utilizada para identificar la inmunización, solo un artículo utilizó el marcador serológico para la hepatitis B (anti-HBs), estimando una prevalencia del 71.6 % (IC 95 %; 62.5 %–79.2 %) de ACS inmunizados^(16)^. Además, dos artículos utilizaron el autoinforme de la realización de este marcador, con prevalencias estimadas del 30,0 %^(32)^ y el 30,2 %^(33)^.

## DISCUSIÓN

El análisis de las evidencias disponibles, tal y como se describe en esta revisión de alcance, puso de manifiesto la escasez de estudios que investiguen de forma exhaustiva la prevalencia global y las tasas de inmunización contra la hepatitis B. Solo dos trabajos incluidos en la muestra contemplaron la evaluación de los marcadores serológicos de la hepatitis B en agentes comunitarios de salud^(15,16)^. Sin embargo, ambos carecen de metodologías basadas en pruebas de laboratorio sensibles para la detección precisa de estos marcadores. Concretamente, el estudio de Amaral et al.^(16)^ utilizó una prueba rápida para identificar el antígeno de superficie de la hepatitis B (HBsAg); sin embargo, el uso exclusivo de este método puede limitar la precisión del diagnóstico, dada la posibilidad de resultados falsos positivos. Se recomendaría la adopción de técnicas confirmatorias, como ensayos inmunoenzimáticos o pruebas moleculares, para garantizar una mayor fiabilidad de los resultados. La literatura científica recomienda que los estudios de prevalencia y cobertura vacunal incluyan la evaluación de los marcadores HBsAg, anti-HBc y anti-HBs, utilizando pruebas más sensibles, a fin de garantizar una evaluación más precisa de la inmunidad poblacional y la circulación del virus^(6,27)^.

Este hallazgo pone de manifiesto la desparametrización y el desconocimiento en relación con el diagnóstico de la hepatitis B y sirve como una importante alerta para reforzar la urgencia de la formación de los profesionales de la salud, la cualificación profesional y la disponibilidad de herramientas de laboratorio más sensibles^(42)^. La práctica clínica basada en la evidencia enfatiza la necesidad de utilizar un panel completo de marcadores serológicos, ya que la ausencia de pruebas completas puede generar resultados falsos positivos o falsos negativos, tratamientos innecesarios y una subestimación de la inmunidad poblacional, lo que dificulta la identificación de individuos susceptibles y la implementación de medidas preventivas eficaces, lo que puede conducir a fallos en la vigilancia epidemiológica^(26)^.

Además, nueve de los artículos analizados evaluaron la seroprevalencia y la inmunización contra la hepatitis B utilizando el autorreporte como método de recopilación de datos^(32,33)^. A pesar de ser una herramienta práctica, de bajo costo y fácil aplicación, especialmente en estudios poblacionales y en contextos donde el acceso a los registros de vacunación es restringido, el autorreporte presenta limitaciones importantes, como el sesgo de memoria, que puede comprometer la confiabilidad de los resultados^(6)^. Una posible justificación para la adopción de este enfoque es la limitación de la financiación para la investigación, ya que la realización de exámenes de laboratorio, aunque más precisa, implica costos significativamente más elevados en comparación con el uso del autorreporte.

Los estudios disponibles indican que complementar el autorreporte con el análisis de registros de vacunación, como la cartilla de vacunación, o con pruebas de laboratorio, como la dosificación de marcadores serológicos, aumenta significativamente la precisión en la evaluación de la situación de vacunación^(3,43)^. Sin embargo, el acceso a la cartilla de vacunación sigue representando un obstáculo para los profesionales de la salud y los investigadores, ya que la población, en general, no tiene la costumbre de considerar este documento como algo esencial que debe almacenarse adecuadamente^(44)^. En este contexto, se hace evidente la necesidad de informatizar el registro de vacunación, aunque esta realidad aún está lejos en muchos escenarios debido a las desigualdades estructurales y digitales que enfrentan las unidades de salud a nivel mundial.

Todos los estudios seleccionados se realizaron en Brasil, muy posiblemente debido a que los ACS son considerados profesionales de la salud^(45)^, lo que demuestra la importancia del Sistema Único de Salud brasileño. En contraste, en otros países, los ACS suelen actuar como voluntarios, reciben una baja remuneración y no cuentan con el debido reconocimiento profesional^(46)^.

Además, esta interpretación está respaldada por la legislación específica de la categoría, que establece directrices fundamentales relacionadas con las condiciones insalubres, la realización de exámenes ocupacionales periódicos y la disponibilidad y el uso adecuado de equipos de protección individual (EPI)^(47)^. Además, los hallazgos refuerzan los esfuerzos continuos del Programa Nacional de Inmunización (PNI) de Brasil, que desempeña un papel crucial en el seguimiento y la mejora de las tasas de inmunización, además de contribuir a la identificación de deficiencias en los sistemas de salud locales^(48)^.

El liderazgo de Brasil en la implementación de estrategias de vacunación y salud pública pone de manifiesto la necesidad de ampliar las investigaciones internacionales, lo que permitiría una comparación más amplia entre diferentes contextos y soluciones globales.

La mayoría de los estudios sobre la hepatitis B en Brasil se han realizado en la región sudeste^(33,36,37,40,41)^, mientras que los estados con mayor prevalencia de la infección, como los de la región noreste^(38,39)^, han estado infrarrepresentados en la literatura científica^(49)^. Esta disparidad pone de manifiesto las desigualdades en el acceso a la atención sanitaria, lo que refleja las desigualdades estructurales que repercuten directamente en la vigilancia epidemiológica y la aplicación de políticas públicas eficaces.

Ante este escenario, es esencial invertir en programas de salud laboral que tengan en cuenta las especificidades de esta categoría profesional, especialmente en regiones históricamente desatendidas. La ampliación de la cobertura vacunal, el fortalecimiento de la vigilancia epidemiológica y la implementación de estrategias de prevención específicas pueden contribuir significativamente a la reducción de las desigualdades y a la mejora de la salud ocupacional.

Aunque los ACS desempeñan un papel fundamental en la promoción de la salud en Brasil, se observa que la mayoría de los estudios disponibles abordan los TAS de manera amplia, sin tener en cuenta las especificidades de estos trabajadores^(15,33,35–39)^. Esta limitación pone de manifiesto una laguna en el conocimiento sobre la actuación específica de los ACS en diferentes contextos regionales.

Además, existe una necesidad urgente de implementar programas de incentivo a la inmunización y verificación de la inmunidad, considerando las disparidades en la cobertura vacunal entre las diversas regiones del país. La desigualdad en la adhesión a las estrategias de vacunación puede comprometer la eficacia de las políticas de salud pública, por lo que es esencial desarrollar enfoques dirigidos a las poblaciones vulnerables y ampliar el papel de los ACS en la movilización comunitaria para la inmunización^(50)^.

Cabe destacar que el enfermero desempeña un papel esencial en la salud de los ACS, contribuyendo a la gestión eficiente, la educación en salud, la administración segura de las vacunas y el monitoreo epidemiológico^(51)^. La actuación de estos profesionales es fundamental para garantizar una alta cobertura de vacunación, prevenir brotes de enfermedades y fortalecer la salud pública^(51)^.

La relación de trabajo entre el enfermero y el agente comunitario de salud es esencial para la eficacia de las acciones de atención primaria de salud, garantizando la implementación de las directrices del Sistema Único de Salud^(52,53)^. Esta colaboración se basa en la supervisión, la coordinación y la colaboración, lo que garantiza que las actividades desarrolladas por los ACS se ajusten a las necesidades de la población y a las políticas públicas de salud^(53)^.

El enfermero desempeña un papel estratégico en la gestión eficiente de los servicios, promoviendo la formación continua de los ACS y garantizando el seguimiento epidemiológico de las condiciones de trabajo. Además, es responsable de la implementación de medidas de bioseguridad, incluida la inmunización, que protege tanto a los profesionales como a la comunidad atendida^(52)^.

La actuación integrada entre enfermeros y ACS fortalece la capilaridad de la atención primaria, ampliando el acceso a los servicios de salud y contribuyendo a la prevención de enfermedades y la promoción de la salud. De este modo, esta relación de trabajo es un pilar fundamental para la mejora de los indicadores de salud y para la equidad en la atención a la población^(52)^.

En este estudio deben tenerse en cuenta algunas limitaciones. Debido a la diversidad de nomenclaturas para los ACS, es posible que algunos estudios no se hayan incluido en la búsqueda, aunque hemos intentado minimizar esta limitación mediante la adición de palabras clave adicionales. Además, cabe señalar que este tipo de revisión no tiene como objetivo evaluar la calidad metodológica de los estudios.

## CONCLUSIÓN

La información recopilada a través de esta revisión de alcance no permitió estimar la prevalencia global de los marcadores serológicos ni de la vacunación entre los ACS, debido a la falta de evidencia científica y a la desparametrización de las medidas de resultado. Es necesario establecer un programa de investigación para subsanar la falta de conocimientos sobre la exposición ocupacional al virus de la hepatitis B entre los ACS, con temas relacionados con las principales exposiciones ocupacionales en diferentes países/ámbitos de actuación, estudios para identificar la prevalencia de marcadores serológicos y de vacunación, y la formación en bioseguridad en los planes de estudio de estos profesionales.

## DISPONIBILIDAD DE DATOS

El conjunto de datos que respaldan las conclusiones de este estudio está disponible previa solicitud al autor correspondiente.
